# TMEM41B is an endoplasmic reticulum Ca^2+^ release channel maintaining naive T cell quiescence and responsiveness

**DOI:** 10.1038/s41421-024-00766-w

**Published:** 2025-03-04

**Authors:** Yuying Ma, Yi Wang, Xiaocui Zhao, Gang Jin, Jing Xu, Zhuoyang Li, Na Yin, Zhaobing Gao, Bingqing Xia, Min Peng

**Affiliations:** 1 State Key Laboratory of Molecular Oncology, Institute for Immunology, Beijing Key Laboratory for Immunological Research on Chronic Diseases, School of Basic Medical Sciences, Tsinghua University, Beijing, China; 2 SXMU-Tsinghua Collaborative Innovation Center for Frontier Medicine, Shanxi Medical University, Taiyuan, Shanxi China; 3 Tsinghua-Peking Center for Life Sciences, Beijing, China; 4 CAS Key Laboratory of Receptor Research, State Key Laboratory of Drug Research, Shanghai Institute of Materia Medica, Chinese Academy of Sciences, Shanghai, China; 5 University of Chinese Academy of Sciences, Beijing, China

**Keywords:** Cell signalling, Autoimmunity

## Abstract

In mammalian cells, endoplasmic reticulum (ER) passively releases Ca^2+^ under steady state, but channels involved remain elusive. Here, we report that TMEM41B, an ER-resident membrane protein critical for autophagy, lipid metabolism, and viral infection, functions as an ER Ca^2+^ release channel. Biochemically, purified recombinant TMEM41B forms a concentration-dependent Ca^2+^ channel in single-channel electrophysiology assays. Cellularly, TMEM41B deficiency causes ER Ca^2+^ overload, while overexpression of TMEM41B depletes ER Ca^2+^. Immunologically, ER Ca^2+^ overload leads to upregulation of IL-2 and IL-7 receptors in naive T cells, which in turn increases basal signaling of JAK-STAT, AKT-mTOR, and MAPK pathways. This dysregulation drives TMEM41B-deficient naive T cells into a metabolically activated yet immunologically naive state. ER Ca^2+^ overload also downregulates CD5, lowering the activation threshold of TMEM41B-deficient T cells and leading to heightened T cell responses during infections. In summary, we identify TMEM41B as a concentration-dependent ER Ca^2+^ release channel, revealing an unexpected role of ER Ca^2+^ in naive T cell quiescence and responsiveness.

## Introduction

Ca^2+^ is a universal second messenger in eukaryotic cells^1^. Endoplasmic reticulum (ER) serves as the primary store for intracellular Ca^2+^, and aberrant levels of Ca^2+^ in ER induce ER stress and contribute to numerous diseases^2–4^. In resting cells, the concentration of Ca^2+^ in ER ([Ca^2+^]_ER_) is maintained in millimolar (mM) range, while cytosolic Ca^2+^ ([Ca^2+^]_cyto_) is in nanomolar (nM) range^5^. This large difference in [Ca^2+^] between ER lumen and cytosol is established by continuous pumping of Ca^2+^ from cytosol into ER by the SERCA family ATPases^6^, which is balanced by either active or passive Ca^2+^ release from ER. Active ER Ca^2+^ release is usually triggered by external stimuli. For example, in activated T cells, T cell receptor (TCR) signaling triggers rapid ER Ca^2+^ release via IP3R channels. This depletion of ER Ca^2+^ store activates the Ca^2+^ sensor STIM1/2, leading to the opening of Ca^2+^ channels on plasma membrane and subsequent Ca^2+^ influx, a process known as store-operated Ca^2+^ entry (SOCE). SOCE is crucial for T cell activation, metabolic reprogramming, and function^7–9^.

Besides active Ca^2+^ release upon stimulation, ER also passively releases Ca^2+^ under steady state. Over 60 years ago, passive and concentration-dependent Ca^2+^ release from ER was observed in mammalian cells^10^. Thapsigargin (TG), an inhibitor of SERCA, induces rapid ER Ca^2+^ release in virtually all metazoan cells by blocking the function of SERCA^11,12^, indicating that ER Ca^2+^ is continuously released into the cytosol under steady-state conditions. Nevertheless, the specific Ca^2+^ channel(s) responsible for steady-state ER Ca^2+^ release remain elusive, posing a longstanding mystery in cell biology^13–15^.

The homeostasis of naive T cells is essential for immunity and tolerance. Following maturation in the thymus, naive T cells exist in a quiescent state in the periphery until activated by cognate antigens. The quiescence of naive T cells is not merely a passive condition but rather an actively maintained state^16–18^. Aside from extrinsic regulation by regulatory T cells and immunosuppressive cytokines^19,20^, the intrinsic regulation of naive T cell quiescence occurs at various levels, including epigenetic, transcriptional, post-transcriptional and signal transduction mechanisms^21^. While significant progress has been made in understanding the critical roles of metabolic reprogramming in activated T cells^22–25^, the metabolic regulation of quiescence in resting naive T cells has received comparatively less attention. Consequently, although it is well-established that naive T cells are metabolically quiescent^22^, the specific mechanisms preserving such metabolic quiescence remain poorly understood^26^.

TMEM41B, a poorly characterized multiple-spanning membrane protein on ER, was initially discovered as an important regulator of autophagy in two independent CRISPR screens^27,28^. Amidst the COVID-19 pandemic, while searching for viral host factors through CRISPR screening, multiple groups independently identified TMEM41B as a pan-flavivirus and pan-coronavirus (including SARS-CoV-2) host factor^29–31^. Biochemically, TMEM41B exhibits phospholipid scramblase activity^32,33^, suggesting potential involvement in lipid metabolism and viral infection^34–36^. Despite these insights, the biochemical nature and physiological function of TMEM41B in vivo remain to be fully understood. In this study, we demonstrate that TMEM41B functions as an ER Ca^2+^ release channel, which plays an indispensable role in maintaining metabolic quiescence and responsiveness of naive T cells.

## Results

### TMEM41B deficiency causes ER Ca^2+^ overload, while overexpression of TMEM41B depletes ER Ca^2+^

**Fig. 1:**
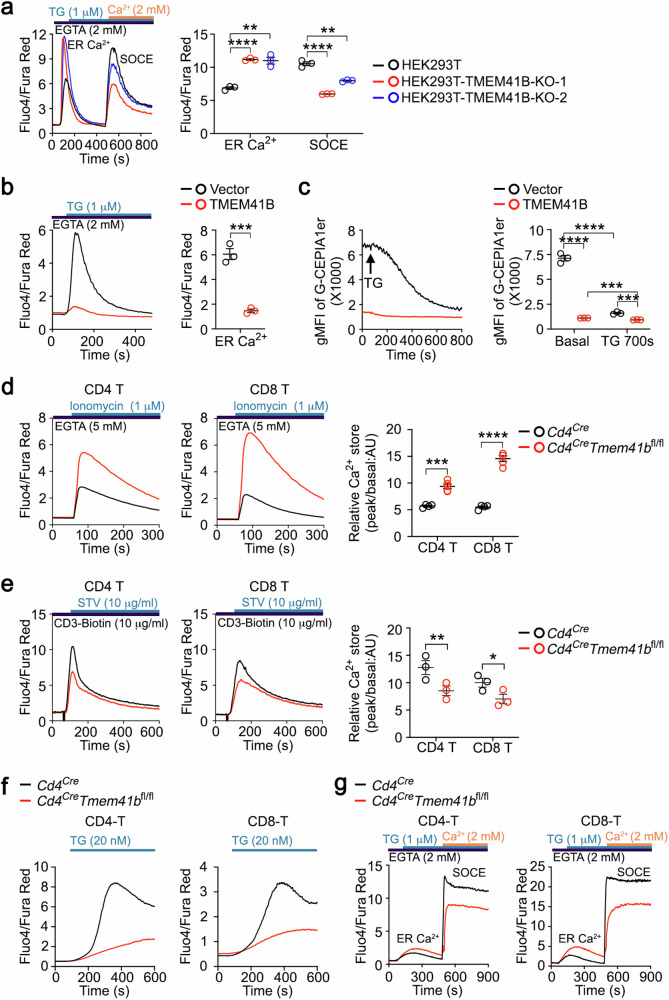
TMEM41B promotes ER Ca^2+^ release. **a** Flow cytometry analysis of ER Ca^2+^ store and SOCE in control and TMEM41B knockout (KO) HEK293T monoclonal cells. Representative plots and statistical analysis from one of three independent experiments are shown (*n* = 3 samples). **b** HEK293T cells were transfected with the indicated plasmids together with a BFP reporter. ER Ca^2+^ store of BFP^high^ HEK293T cells was examined by flow cytometry. Representative plots and statistical analysis from one of three independent experiments are presented (*n* = 3 samples). **c** HEK293T-G-CEPIA1er cells were transfected with the indicated plasmids together with a BFP reporter. ER Ca^2+^ store of BFP^high^ HEK293T-G-CEPIA1er cells was examined by flow cytometry before and after TG (1 μM) treatment. Representative plots and statistical analysis from one of three independent experiments are presented (*n* = 3 samples). **d** Flow cytometry analysis of ER Ca^2+^ store in T cells with the indicated genotypes. Representative plots and statistical analysis are shown (*n* = 4 mice). **e** Flow cytometry analysis of αCD3-induced Ca^2+^ influx in T cells with the indicated genotypes. Representative plots and statistical analysis are shown (*n* = 3 mice). **f** Flow cytometry analysis of 20 nM TG-induced Ca^2+^ influx (SOCE) in T cells with the indicated genotypes. Representative plots are shown (*n* = 4 mice). **g** Flow cytometry analysis of ER Ca^2+^ store and SOCE in T cells with the indicated genotypes. Representative plots are shown (*n* = 3 mice). The control group results of **a**–**d** were utilized in our previous study^37^. Data represent mean ± SEM; one-way ANOVA in **a** and **c**; two-tailed unpaired *t*-test in **b**, **d** and **e**; *P < 0.05, **P < 0.01, ***P < 0.001, ****P < 0.0001.

In a genome-wide CRISPR screening for regulators of ER Ca^2+^, we revealed a role of VMP1 in ER Ca^2+^ homeostasis^37^. Since TMEM41B and VMP1 are related, we explored the potential role of TMEM41B in ER Ca^2+^ regulation in this study. In a standard SOCE assay, TMEM41B deficiency resulted in an increase in ER Ca^2+^ levels and an attenuated SOCE in HEK293T cells (Fig. 1a), an observation confirmed in two independent TMEM41B-knockout HEK293T monoclonal cell clones (Supplementary Fig. S1a). These data indicate that TMEM41B is required for ER Ca^2+^ release and optimal SOCE in HEK293T cells.

Importantly, overexpression of TMEM41B resulted in nearly complete depletion of ER Ca^2+^ in HEK293T cells (Fig. 1b). To directly monitor ER Ca^2+^ levels, we utilized a HEK293T cell line that stably expressed the ER Ca^2+^ sensor G-CEPIA1er^38^. In HEK293T-G-CEPIA1er cells, steady-state ER Ca^2+^ levels were almost completely depleted upon TMEM41B overexpression, and minimal ER Ca^2+^ release was observed after TG treatment (Fig. 1c). These findings demonstrate that Ca^2+^ in the ER is almost entirely released upon TMEM41B overexpression.

To extend these findings to primary T cells, we generated T cell-specific *Tmem41b* knockout mice (*Cd4*^*Cre*^*Tmem41b*^*fl/fl*^) through crossing *Tmem41b* floxed mice with *Cd4*^*Cre*^ transgenic mice (Supplementary Fig. S1b). *Tmem41b* knockout was confirmed by PCR analysis (Supplementary Fig. S1c, d). As expected, ER Ca^2+^ levels were increased in TMEM41B-deficient naive T cells compared to control naive T cells (Fig. 1d). Additionally, Ca^2+^ influx, either triggered by CD3 cross-linking or TG treatment, was attenuated in TMEM41B-deficient T cells (Fig. 1e, f). This is consistent with the increased ER Ca^2+^ levels observed in these cells (Fig. 1d), as the extent of SOCE is determined by the residual Ca^2+^ in the ER. In the standard SOCE assay, TMEM41B-deficient T cells also demonstrated increased ER Ca^2+^ levels and diminished SOCE (Fig. 1g).

Collectively, these data establish that TMEM41B is required and sufficient for ER Ca^2+^ release.

### TMEM41B mediates Ca^2+^ transport across membranes

**Fig. 2:**
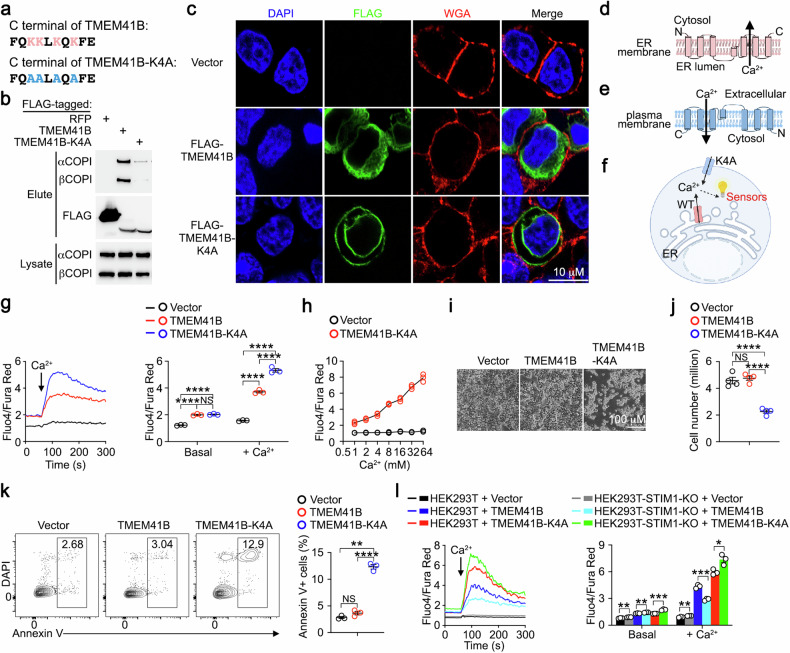
TMEM41B mediates Ca^2+^ transport across membranes. **a** C-terminal amino acid sequence of TMEM41B and TMEM41B-K4A. Lysine residues in Golgi-to-ER retrieval motif are labeled in red and the mutated alanine residues are labeled in blue. **b** The interactions between RFP, TMEM41B or TMEM41B-K4A with COPI were examined by co-immunoprecipitation. Representative data from one of three independent experiments are shown. The first lane result was utilized in our previous study^37^. **c** Immunofluorescence examination of subcellular localization of FLAG-tagged (N-terminal) TMEM41B and TMEM41B-K4A. Plasma membrane was labeled by WGA. Representative data from one of three independent experiments are shown. **d** Putative topology of TMEM41B on ER membrane. **e** Putative topology of TMEM41B-K4A on plasma membrane. **f** Putative Ca^2+^ transport mediated by TMEM41B on ER membrane and TMEM41B-K4A on plasma membrane. **g** Flow cytometry analysis of Ca^2+^ influx in HEK293T cells transfected with the indicated plasmids. CaCl_2_ was added at 60 s to achieve the final concentration of 8 mM to induce Ca^2+^ influx. Representative plots and statistical analysis from one of three independent experiments are shown (*n* = 3 samples). **h** Flow cytometry analysis of Ca^2+^ influx induced by varying concentrations of CaCl_2_ in HEK293T cells transfected with either an empty vector or TMEM41B-K4A. Representative statistical analysis from one of three independent experiments is shown (*n* = 3 samples). **i**, **j** Survival of HEK293T cells transfected with the indicated plasmids (48 h post transfection). Representative images (**i**) and statistical analysis (**j**) from one of three independent experiments are shown (*n* = 4 samples). **k** HEK293T cells were transfected with the indicated plasmids, and apoptosis was assessed via flow cytometry 24 h post transfection. Representative plots and statistical analysis from one of three independent experiments are shown (*n* = 3 samples). **l** Flow cytometry analysis of Ca^2+^ influx in control and STIM1-knockout (KO) HEK293T cells transfected with the indicated plasmids. CaCl_2_ was added at 60 s to achieve the final concentration of 8 mM to induce Ca^2+^ influx. Representative plots and statistical analysis from one of three independent experiments are shown (*n* = 3 samples). The control group results of **g** and **h** were utilized in our previous study^37^. Data represent mean ± SEM; one-way ANOVA in **g**, **j** and **k**; two-tailed unpaired *t*-test in **l**; *P < 0.05, **P < 0.01, ***P < 0.001, ****P < 0.0001, NS not significant.

TMEM41B is an ER-resident membrane protein^27,28^, and it is technically challenging to measure Ca^2+^ transport across the ER membrane. To address this, we redirected TMEM41B to the plasma membrane using a strategy reported previously^37^. TMEM41B possesses a lysine-rich Golgi-to-ER retrieval motif at its C-terminus (Fig. 2a), which interacts with COPI complex, facilitating the retro-transport of membrane proteins from the *trans*-Golgi back to the ER^39^. When lysine residues within this Golgi-to-ER retrieval motif were mutated to alanine (TMEM41B-K4A) (Fig. 2a), the resulting mutant exhibited reduced COPI binding compared to its wild-type (WT) counterpart (Fig. 2b). Unlike WT TMEM41B exhibiting typical ER localization, TMEM41B-K4A exhibited plasma membrane localization in addition to ER localization (Fig. 2c).

According to the topology of membrane proteins, the luminal side of TMEM41B should face extracellularly when expressed on the plasma membrane (Fig. 2d, e). Given that extracellular Ca^2+^ concentrations are high (~mM range) while cytosolic Ca^2+^ concentrations are low (~nM range), plasma membrane-targeted TMEM41B-K4A is expected to induce Ca^2+^ influx if it is capable of channeling Ca^2+^ in the presence of electrochemical gradients of Ca^2+^ (Fig. 2f). In HEK293T cells transfected with empty vector, Ca^2+^ supplementation induced negligible Ca^2+^ influx (Fig. 2g), consistent with the absence of concentration-dependent Ca^2+^ channels on the cell surface in mammalian cells^40^. However, in cells overexpressing WT TMEM41B, a small Ca^2+^ influx was induced by Ca^2+^ supplementation, whereas robust Ca^2+^ influx was observed in cells overexpressing the plasma membrane-targeted TMEM41B-K4A (Fig. 2g). Importantly, the Ca^2+^ influx in cells overexpressing TMEM41B-K4A was concentration-dependent (Fig. 2h). Prolonged Ca^2+^ influx is known to trigger cell death^11,12^. Consistently, HEK293T cells overexpressing plasma membrane-targeted TMEM41B-K4A exhibited increased apoptosis compared to control cells (Fig. 2i–k).

To investigate whether the observed Ca^2+^ influx in HEK293T cells overexpressing TMEM41B or TMEM41B-K4A was SOCE, we repeated these experiments in STIM1- or ORAI1-deficient HEK293T cells, which are incapable of inducing SOCE^37^. Ca^2+^ influx induced by TMEM41B overexpression was abolished in STIM1- or ORAI1-deficient HEK293T cells, while TMEM41B-K4A-induced Ca^2+^ influx remained unaffected (Fig. 2l; Supplementary Fig. S1e). This demonstrates that Ca^2+^ influx induced by TMEM41B overexpression is SOCE, due to its depletion of ER Ca^2+^, whereas TMEM41B-K4A-induced Ca^2+^ influx is not mediated by SOCE.

Together, these findings demonstrate that TMEM41B is able to mediate Ca^2+^ transport across membranes, either by acting as a Ca^2+^ channel itself or by facilitating the opening of Ca^2+^ channel(s) other than ORAI1.

### TMEM41B forms a Ca^2+^-permeable channel

To investigate whether TMEM41B functions as a Ca^2+^ channel, we conducted single-channel electrophysiology assays using purified recombinant TMEM41B (Supplementary Fig. S1f–h). Although the molecular weight of TMEM41B was 25 kDa on denatured gels (Supplementary Fig. S1g, h), TMEM41B appeared significantly larger than the monomeric form on native gels (Supplementary Fig. S1i, j), suggesting that purified TMEM41B exists as oligomers, a typical feature of ion channels.

**Fig. 3:**
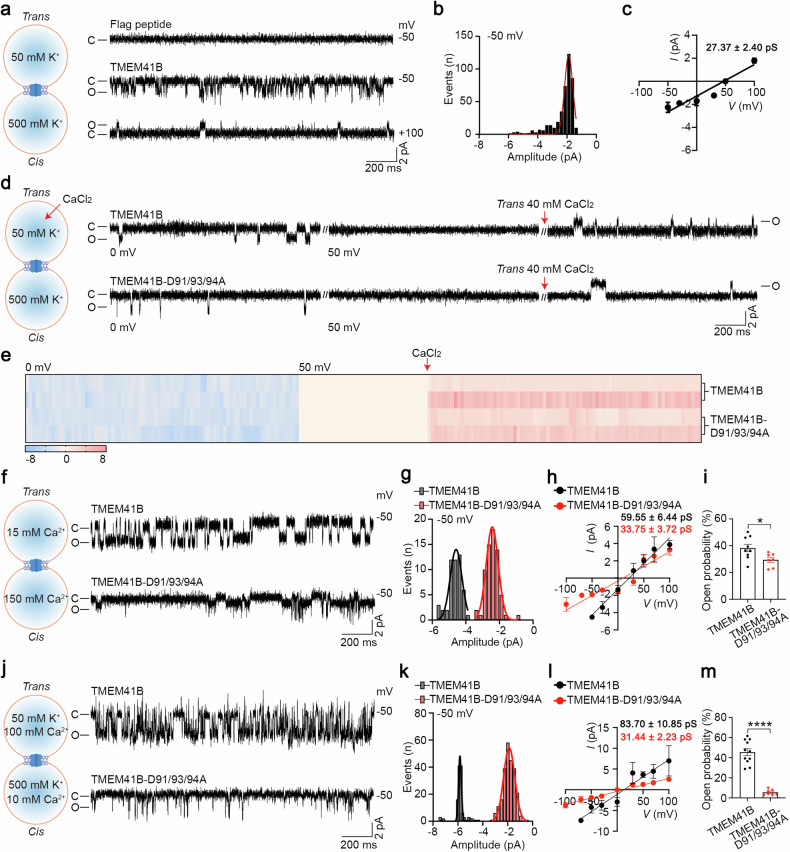
TMEM41B forms a Ca^2+^-permeable channel. **a** Left, the cartoon depicts the planar lipid bilayer work station, while the recording solutions on both sides are displayed in the panel. Right, representative single-channel currents of TMEM41B or Flag peptide at the indicated voltage. “C” means closed; “O” means open. **b** All-point current histograms for the trace in **a**. **c** I–V curve of TMEM41B in solution of **a** (*n* ≥ 5 independent experiments). **d** Ca^2+^ currents of TMEM41B and TMEM41B-D91/93/94 A recorded in K^+^ solutions. Red arrow indicates that the CaCl_2_ solutions were added (*n* = 3 independent experiments). **e** The heat map shows an overview of the current events in **d**. The blue sections represent the current amplitude at 0 mV, the beige sections indicate the current amplitude at the equilibrium voltage, and the red sections correspond to the current amplitude after the addition of Ca^2+^. Each row represents an individual case. **f** Left, the recording solutions in *cis* and *trans* sides. Right, representative single-channel currents of TMEM41B and TMEM41B-D91/93/94 A at –50 mV in pure Ca^2+^ solutions. **g** All-point current histograms for the trace in **f**. **h** I–V curve of TMEM41B and TMEM41B-D91/93/94 A in solution of **f**. **i** Open probability of TMEM41B and TMEM41B-D91/93/94 A in solution of **f** (*n* ≥ 5 independent experiments). **j** Left, the recording solutions in *cis* and *trans* sides. Right, representative single-channel currents of TMEM41B and TMEM41B-D91/93/94 A at –50 mV in K^+^/Ca^2+^ mixture solutions. **k** All-point current histograms for the trace in **j**. **l** I–V curve of TMEM41B and TMEM41B-D91/93/94 A in solution of **j**. **m** Open probability of TMEM41B and TMEM41B-D91/93/94 A in solution of **j**. Data represent mean ± SEM; two-tailed unpaired *t*-test in **i** and **m**; *P < 0.05, ****P < 0.0001.

Single-channel currents of TMEM41B were recorded using planar lipid bilayer experiments (Supplementary Fig. S1k). We successfully recorded single-channel currents of TMEM41B with a conductance of 27.37 + 2.40 pS (mean ± SEM) (Fig. 3a–c). As a control, the elution buffer (containing FLAG peptide) did not exhibit detectable currents (Fig. 3a). In an asymmetric 50 mM:500 mM KCl solution, TMEM41B displayed a reverse potential of 52.77 mV, resulting in an estimated P_K+_/P_Cl–_ of 31.77, indicating that TMEM41B is a cation-selective channel (Fig. 3c).

To explore the permeability of TMEM41B to Ca^2+^, Ca^2+^ currents were analyzed. After a stable current was recorded (Fig. 3d), the membrane potential was adjusted to the K^+^ equilibrium potential (50 mV) to eliminate K^+^ currents. Subsequently, Ca^2+^ was added to the *trans* side to assess TMEM41B’s permeability to Ca^2+^. Inward step-like signals were observed when 40 mM (final concentration) Ca^2+^ was added to the *trans* side (Fig. 3d, e), suggesting that Ca^2+^ could pass through TMEM41B channels.

Further investigation of TMEM41B channel activity was conducted in a pure calcium solution (150 mM:15 mM). After the formation of stable planar lipid bilayers, obvious single-channel currents appeared following the addition of purified TMEM41B proteins (Fig. 3f). The single-channel current amplitude of TMEM41B proteins acquired by the Gauss fit was approximately –4.58 pA at a –50 mV potential (Fig. 3g). The current-potential (I–V) curve of these single-channel currents was plotted, resulting in a 59.55 ± 6.44 pS Ca^2+^ conductance (Fig. 3h).

Additionally, the addition of asymmetric Ca^2+^ (100 mM:10 mM) into the bath solution caused the reversal potential of TMEM41B to shift from 52.77 mV to 13.04 mV (Fig. 3j–l). This substantial shift of reversal potential suggests that TMEM41B could be permeable to Ca^2+^. Importantly, the conductance of TMEM41B increased to 83.70 ± 10.85 pS after adding CaCl_2_ to the bath solution (Fig. 3l).

To identify key residues of TMEM41B involved in Ca^2+^ channeling, we individually mutated all negatively charged amino acids (aspartic acid and glutamic acid) on the ER lumen side of TMEM41B. Employing a rescue system, where TMEM41B and mutants were re-expressed in TMEM41B-deficient primary T cells to reverse ER Ca^2+^ overload caused by TMEM41B deficiency, we found that none of the single amino acid mutants exhibited loss of function in our reconstitution assay (data not shown). However, simultaneous mutation of aspartate residues at positions 91, 93, and 94 (D91/93/94) to alanine (D91/93/94 A) resulted in a partial loss of function in TMEM41B-mediated ER Ca^2+^ release (Supplementary Fig. S1l, m).

Subsequently, the D91/93/94 A mutant was purified and recorded accordingly. Electrophysiological results revealed that the D91/93/94 A mutant could form a cation channel (*E*_*rev*_ = 46.96 mV; P_K+_/P_Cl–_ = 15.38) (Supplementary Fig. S1n–p) and is permeable to Ca^2+^ (Fig. 3d, e), similar to WT TMEM41B. While the D91/93/94 A mutant remained permeable to Ca^2+^, its conductance in a pure calcium solution decreased from 59.55 ± 6.44 pS to 33.75 ± 3.72 pS compared to the WT TMEM41B (Fig. 3f–h). Consistent results were also observed in the K^+^/Ca^2+^ mixture solution (*E*_*rev*_ = 12.81 mV; P_K+_/P_Ca2+_ = 1.73) (Fig. 3j–l). Moreover, the open probability of the D91/93/94 A mutant significantly decreased compared to WT TMEM41B in the above two solutions (Fig. 3i, m). Thus, D91/93/94 are critical for Ca^2+^ permeability of the TMEM41B channel.

Finally, the influence of Ca^2+^ on TMEM41B channel was evaluated using a titration strategy. Following the incorporation of channels into membranes, Ca^2+^ (0 mM, 1 mM, 2 mM, 5 mM, 10 mM CaCl_2_) was sequentially titrated into the *cis* side at the indicated voltages, to assess the effect of Ca^2+^ on the existing current. While additional Ca^2+^ did not alter the open probability of the TMEM41B channel, it notably increased the amplitude of the channel, particularly saturating around 2 mM concentrations (Supplementary Fig. S1q–t). In contrast, the addition of Ca^2+^ failed to increase the channel activity (including amplitude and open probability) of the D91/93/94 A mutant under the saturated concentration (2 mM) (Supplementary Fig. S1q, s, u). Thus, D91/93/94 determine the amplitude but not the open probability of the TMEM41B channel. These results underscore the role of Ca^2+^ concentration in modulating the Ca^2+^ permeability of TMEM41B.

These data collectively demonstrate that TMEM41B forms a Ca^2+^ channel, and the activity of this channel depends on Ca^2+^ concentration.

### TMEM41B maintains metabolic quiescence of naive T cells

Deletion of *Tmem41b* using *Cd4*^*Cre*^ did not affect T cell development in the thymus (Supplementary Fig. S2a–c). While a slight reduction in mature CD8 T cells was observed in the secondary lymphoid organs of *Cd4*^*Cre*^*Tmem41b*^*fl/fl*^ mice (Supplementary Fig. S2d), apoptosis of TMEM41B*-*deficient CD8 T cells remained unchanged (Supplementary Fig. S2e, f). The observed decrease in CD8 T cell numbers is likely attributed to heightened ER stress in these cells (Supplementary Fig. S2g, h). However, unlike VMP1*-*deficient T cells, TMEM41B*-*deficient T cells did not exhibit mitochondrial Ca^2+^ overload (Supplementary Fig. S2i, j), a phenomenon that leads to massive apoptosis in VMP1*-*deficient T cells^37^. Thus, TMEM41B appears to play a distinct role in T cells compared to VMP1.

**Fig. 4:**
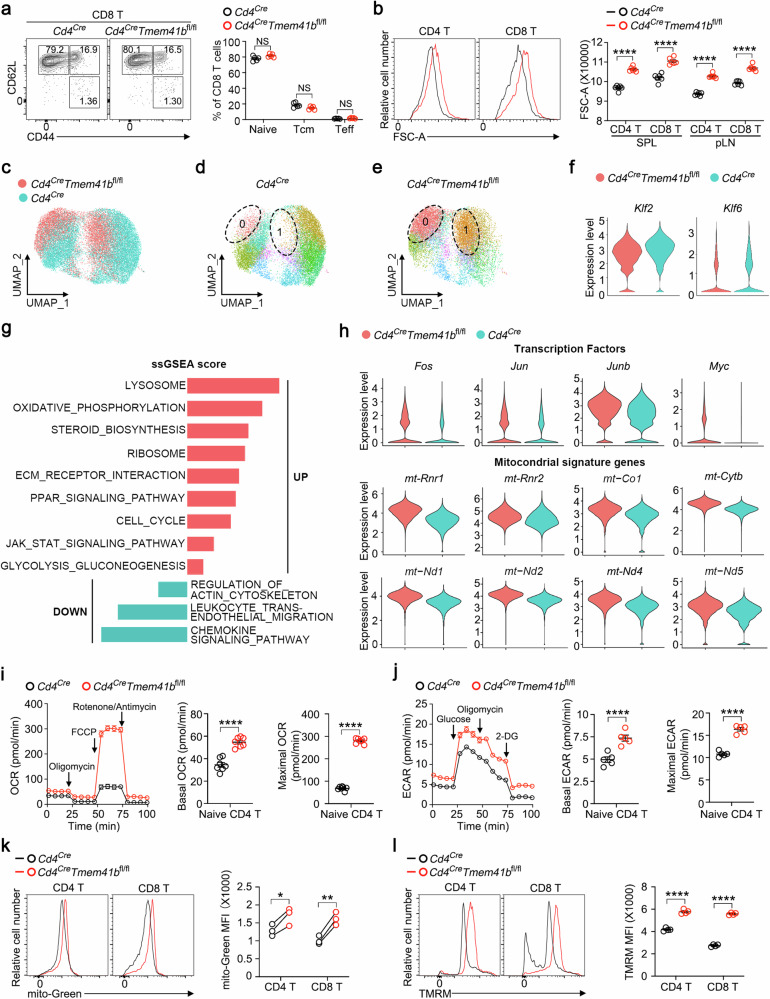
TMEM41B maintains naive T cell quiescence. **a** Flow cytometry analysis of activation status (CD44 vs CD62L) of CD8 T cells from peripheral lymph nodes (pLN) of control (*Cd4*^*Cre*^) and TMEM41B-deficient (*Cd4*^*Cre*^*Tmem41b*^*fl/fl*^) mice (6*–*8 weeks old). Representative plots and statistical analysis are shown (*n* = 5 mice). **b** Flow cytometry analysis of the cell size of control and TMEM41B-deficient T cells from spleen (SPL) and pLN. Representative plots and statistical analysis are shown (*n* = 6 mice). **c** A merged UMAP plot of naive T cells (TCRβ^+^CD44^–^CD62L^+^CD25^–^) from both control and TMEM41B-deficient mice, generated through scRNA-seq to visualize cellular heterogeneity. Each dot represents an individual cell, with red dots indicating TMEM41B-deficient T cells and green dots representing control WT T cells. **d**, **e** UMAP plots illustrating the distribution of control naive T cells (**d**) and TMEM41B-deficient naive T cells (**e**), highlighting cells in clusters 0 and 1. Each dot represents an individual cell. Each cluster is represented by a distinct color. **f** Villon plots displaying gene expression levels in control and TMEM41B-deficient naive T cells. **g** Gene-set enrichment analysis (GSEA) of upregulated and downregulated genes in TMEM41B-deficient naive T cells compared to control naive T cells using the single-sample GSEA (ssGSEA) method. The ssGSEA score reflects the degree to which a gene set is overrepresented (positive score) or underrepresented (negative score) in the TMEM41B-deficient group compared to controls, indicating potential functional pathways affected by TMEM41B deficiency. **h** Villon plots of gene expression in control and TMEM41B-deficient naive T cells. **i** OCRs of control and TMEM41B-deficient naive CD4 T cells were measured with a Mito stress test kit. Representative plots and calculated basal and maximal OCRs from one of three independent experiments are shown (*n* = 7 replicates for each group). **j** ECARs of control and TMEM41B-deficient naive CD4 T cells were measured with a Glycolysis stress test kit. Representative plots and calculated basal and maximal ECARs from one of three independent experiments are shown (*n* = 5 replicates for each group). **k** Flow cytometry analysis of mitochondrial mass of naive T cells with the indicated genotypes. Representative plots and statistical analysis are shown (*n* = 3 mice). **l** Flow cytometry analysis of mitochondrial membrane potential of naive T cells with the indicated genotypes. Representative plots and statistical analysis are shown (*n* = 4 mice). Data represent mean ± SEM; two-tailed unpaired *t*-test in **a**, **b**, **i**, **j** and **l**; paired *t*-test in **k**; *P < 0.05, **P < 0.01, ****P < 0.0001, NS not significant.

Flow cytometry analysis revealed no discernible differences in T cell activation states between TMEM41B-deficient and WT T cells (Fig. 4a; Supplementary Fig. S3a, b). However, TMEM41B-deficient naive T cells exhibited enlarged sizes compared to control naive T cells (Fig. 4b), indicating heightened anabolic activities.

To elucidate the underlying mechanisms, we conducted single-cell RNA sequencing (scRNA-seq) of naive T cells (TCRβ^+^CD62L^+^CD44^–^CD25^–^) isolated from control and TMEM41B-deficient mice. In concordance with the flow cytometry analysis (Fig. 4a; Supplementary Fig. S3a, b), both WT and TMEM41B-deficient naive T cells did not express *Cd44* but displayed high expression of *Sell* (CD62L) (Supplementary Fig. S3c), confirming that the sorted cells were naive T cells. Uniform Manifold Approximation and Projection (UMAP) analysis revealed largely non-overlapping cluster distributions between control and TMEM41B-deficient naive T cells (Fig. 4c), indicating distinct cellular states. Based on disparities in transcriptional profiles, cells, including both WT and TMEM41B-deficient naive T cells, can be clustered into 13 distinct groups (Supplementary Fig. S3d, e). Notably, > 85% of cells in clusters 0 and 1 were TMEM41B-deficient naive T cells, compared to only ~10% of control naive T cells in these two clusters (Supplementary Fig. S3f). Consistently, separate UMAP analysis revealed that the majority of TMEM41B-deficient naive T cells distributed within clusters 0 and 1, whereas control naive T cells were almost absent from these two clusters (Fig. 4d, e). Interestingly, cells in both cluster 0 (CD4^+^) and cluster 1 (CD8^+^) expressed high levels of genes related to mitochondrial activity, including *mt-Rnr1*, *mt-Rnr2*, *mt-Nd1*, *Ndufa13*, *Ndufa6*, *Ndufa5* and *Ndufa11* (Supplementary Fig. S3d, g).

Despite being phenotypically naive (CD62L^+^CD44^–^) (Fig. 4a; Supplementary Fig. S3a–c), TMEM41B-deficient naive T cells displayed downregulation of transcription factors associated with T cell quiescence, including *Klf2* and *Klf6*^41^ (Fig. 4f). Importantly, pathways associated with mitochondrial metabolism, glycolysis, ribosome biogenesis, amino acid metabolism, PPAR signaling, and JAK-STAT signaling were enriched in TMEM41B-deficient naive T cells (Fig. 4g; Supplementary Fig. S3h, i). Specifically, genes conventionally associated with T cell activation, such as *Fos*, *Jun*, *Junb*, and *Myc*, along with mitochondrial genes, such as *mtRnr1*, *mtRnr2*, *mt-Nd1*, *mt-Nd2*, *mt-Nd4*, *mt-Nd5*, *mt-Co1* and *mt-Cytb*, were upregulated in TMEM41B-deficient naive T cells (Fig. 4h; Supplementary Fig. S3j). These transcriptional changes suggest that, although TMEM41B-deficient naive T cells are not activated immunologically, they are in a metabolically active state. Indeed, metabolic assays revealed a significant increase in both the oxygen consumption rate (OCR) and extracellular acidification rate (ECAR) in TMEM41B-deficient naive T cells (Fig. 4i, j), underscoring their heightened metabolic activity in a non-activated state. Consistent with increased OCR, TMEM41B-deficient T cells displayed increased mitochondrial mass and mitochondrial membrane potential (Fig. 4k, l), alongside increased reactive oxygen species (ROS) levels (Supplementary Fig. S3k).

Collectively, these findings demonstrate that TMEM41B deficiency propels naive T cells into a metabolically activated yet immunologically naive state, a phenomenon not reported previously (See Discussion).

### TMEM41B represses IL-2 and IL-7 signaling in naive T cells via Ca^2+^ channel activity

**Fig. 5:**
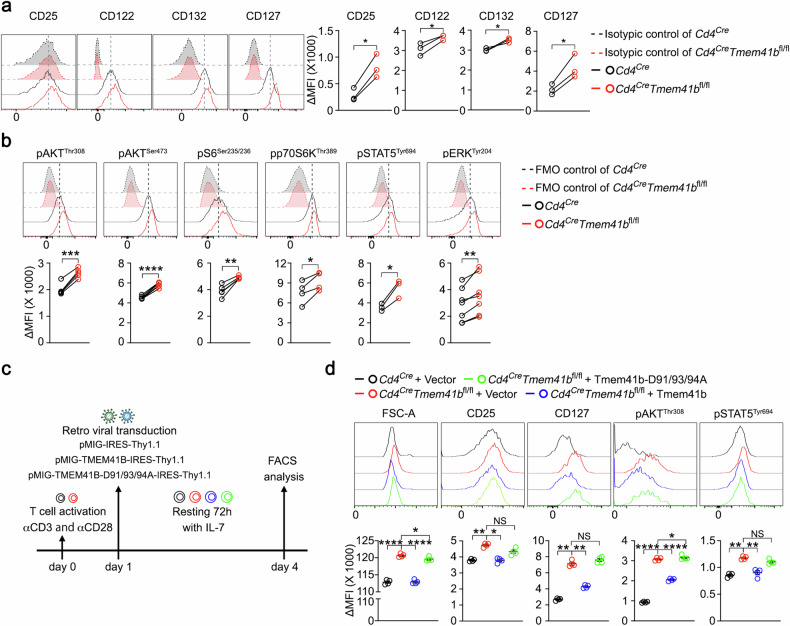
TMEM41B represses basal IL-2 and IL-7 receptor signaling in naive T cells via Ca^2+^ channel activity. **a** Flow cytometry analysis was conducted to assess the expression levels of the indicated proteins in naive CD8 T cells isolated from pLN of control (*Cd4*^*Cre*^) and TMEM41B-deficient (*Cd4*^*Cre*^*Tmem41b*^*fl/fl*^) mice. ΔMFI (mean fluorescence intensity) represents the MFI of the target marker after subtracting the MFI of the isotype control. Representative plots and statistical analysis are shown (*n* = 3 mice). **b** Flow cytometry analysis was conducted to assess the phosphorylation levels of the indicated proteins in naive CD8 T cells isolated from pLN of control and TMEM41B-deficient mice. Fluorescence minus one (FMO) control was used to ensure accurate measurement. ΔMFI represents the MFI of the target marker after subtracting the MFI of the FMO control. Representative plots and statistical analysis are shown (*n* = 3–8 mice). **c** Experimental design for rescuing phenotypes observed in TMEM41B-deficient T cells by overexpressing either WT TMEM41B or mutant TMEM41B-D91/93/94 A. **d** Activated CD4 T cells were transduced with retroviral constructs as indicated. Flow cytometry analysis was performed 72 h post transduction to measure cell size, CD25/CD127 expression, and phosphorylation levels of AKT and STAT5. ΔMFI represents the MFI of the target marker after subtracting the MFI of the FMO control. Representative plots and statistical analysis from one of three independent experiments are shown (*n* = 4 samples). Data represent mean ± SEM; two-tailed paired *t*-test in **a** and **b**; one-way ANOVA in **d**; *P < 0.05, **P < 0.01, ***P < 0.001, ****P < 0.0001, NS not significant.

To unravel the mechanisms underlying the metabolic activation, but not immunological activation, of TMEM41B-deficient naive T cells, we screened receptors implicated in promoting metabolism but unable to induce T cell activation independently. These receptors include IL-2Rα (CD25), IL-2Rβ (CD122), IL-2Rγ (CD132), and IL-7R (CD127). Our results revealed consistent elevation of both IL-2 receptors (CD25, CD122, and CD132) and IL-7R in TMEM41B-deficient naive T cells compared to control naive T cells (Fig. 5a; Supplementary Fig. S4a, b). Subsequent examination of downstream signaling events originating from IL-2 and IL-7 receptors, such as the JAK-STAT, AKT-mTOR, and MAPK pathways, revealed increased phosphorylation of AKT, S6, p70S6K, STAT5, and ERK in TMEM41B-deficient naive T cells relative to control naive T cells (Fig. 5b; Supplementary Fig. S4c). These findings indicate enhanced signaling from IL-2 and/or IL-7 receptors in TMEM41B-deficient naive T cells in the absence of T cell activation.

To elucidate the relationship between ER Ca^2+^ overload and increased IL-2/IL-7 receptor signaling in TMEM41B-deficient T cells, we performed a rescue experiment using WT and D91/93/94 A mutant TMEM41B, which exhibited reduced Ca^2+^ channel activity (Fig. 3). In these experiments, both control and TMEM41B-deficient T cells were first activated to facilitate retroviral transduction of WT TMEM41B or the D91/93/94 A mutant. This was followed by a 3-day rest period with IL-7 treatment, allowing the cells to transition into a relatively quiescent state (Fig. 5c). Under this condition, the upregulation of CD25 and CD127, enhanced AKT and STAT5 signaling, as well as the enlarged cell size of TMEM41B-deficient T cells, were all reversed by the WT TMEM41B but not the D91/93/94 A mutant (Fig. 5d; Supplementary Fig. S4d). These findings suggest that these changes are associated with ER Ca^2+^ dysregulation.

Cell size is primarily determined by protein content, with the mTORC1 pathway playing critical roles in T cell biosynthesis and metabolism^42^. Given the activation of mTORC1 observed in TMEM41B-deficient T cells (Fig. 5b; Supplementary Fig. S4c), we investigated whether inhibiting mTORC1 could rescue the metabolic phenotypes of these cells. We generated *Cd4*^*Cre*^*Tmem41b*^*fl/fl*^*Rptor*^*fl/fl*^ mice by crossing *Cd4*^*Cre*^*Tmem41b*^*fl/fl*^ mice with *Rptor* flox mice to deplete RAPTOR (Supplementary Fig. S5a), the essential component of the mTORC1^43^. Notably, RAPTOR deficiency significantly reversed the enlarged size of TMEM41B-deficient T cells (Supplementary Fig. S5b, c). Moreover, the increased OCR and ECAR in TMEM41B-deficient T cells were also partially mitigated by RAPTOR deficiency (Supplementary Fig. S5d–g). These findings demonstrate that heightened mTORC1 signaling contributes to the metabolic activation of TMEM41B-deficient T cells. However, the incomplete rescue by RAPTOR deficiency suggests that other pathways, such as STAT5 or ERK, also contribute to the metabolic activation of TMEM41B-deficient T cells.

Collectively, these data demonstrate that ER Ca^2+^ overload in TMEM41B-deficient T cells, through yet unidentified mechanism(s), upregulates IL-2/IL-7 receptors, which consequently leads to constitutive JAK-STAT, AKT-mTOR, and MAPK signaling, ultimately resulting in the metabolic activation of TMEM41B-deficient naive T cells.

### TMEM41B represses T cell responsiveness in part via CD5

**Fig. 6:**
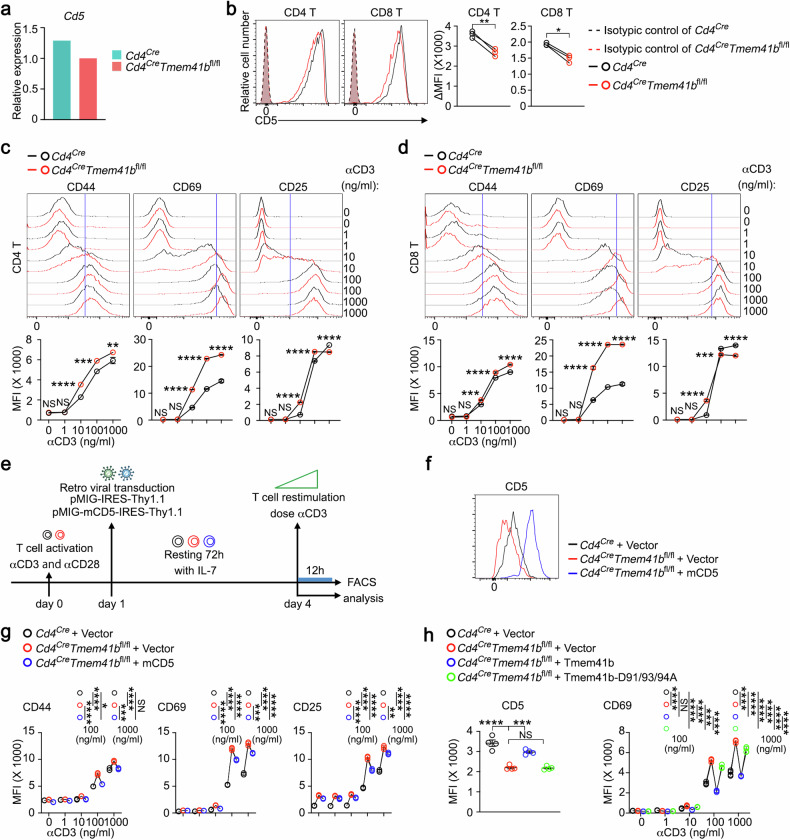
TMEM41B represses T cell responsiveness in part via CD5. **a** Relative mRNA levels of *Cd5* in naive T cells from control (*Cd4*^*Cre*^) and TMEM41B-deficient (*Cd4*^*Cre*^*Tmem41b*^*fl/fl*^) mice, as determined from scRNA-seq data. **b** Flow cytometry analysis of CD5 expression in naive T cells isolated from pLN of control and TMEM41B-deficient mice. ΔMFI represents the MFI of the target marker after subtracting the MFI of the isotype control. Representative plots and statistical analysis are shown (*n* = 3 mice). **c**, **d** Flow cytometry analysis of the upregulation of CD44, CD69 and CD25 in CD4 T cells (**c**) and CD8 T cells (**d**) following stimulation with varying doses of αCD3 antibody. Representative flow cytometry plots (upper) and corresponding statistical analysis (lower) from one of three independent experiments are shown (*n* = 3 samples). **e** Schematic representation of the experimental design for rescuing CD5-related phenotypes in TMEM41B-deficient T cells. **f** Flow cytometry analysis of CD5 expression on CD8 T cells transduced with the indicated constructs. Representative flow cytometry plots are shown. **g** Flow cytometry analysis of CD44, CD69 and CD25 expression on CD8 T cells transduced with the indicated constructs upon αCD3 stimulation. Representative statistical analysis from one of three independent experiments is shown (*n* = 3 samples). **h** Flow cytometry analysis of CD5 (before αCD3 restimulation) and CD69 (after αCD3 restimulation) expression on CD8 T cells transduced with the indicated constructs. Representative statistical analysis from one of three independent experiments is shown (*n* = 4 samples). Data represent mean ± SEM; paired *t*-test in **b**; two-way ANOVA in **c** and **d**; one-way ANOVA in **g** and **h**; *P < 0.05, **P < 0.01, ***P < 0.001, ****P < 0.0001, NS not significant.

Upon analyzing scRNA-seq data, we observed that *Cd5*, a negative regulator of TCR signaling^44^, was downregulated in TMEM41B-deficient naive T cells (Fig. 6a). This finding was further validated at the protein level through flow cytometry (Fig. 6b). Given CD5’s role in repressing TCR signaling, we speculated that TMEM41B-deficient T cells might be hyperreactive to antigen stimulation. Indeed, the upregulation of CD69 was more pronounced in TMEM41B-deficient T cells compared to control T cells, particularly at lower doses of CD3 cross-linking (Fig. 6c, d). Additionally, there was a trend toward higher expression of CD44 and CD25 in TMEM41B-deficient T cells compared to control T cells, especially at lower doses of CD3 cross-linking (Fig. 6c, d).

To explore whether reduced CD5 expression drives the hyperresponsiveness of TMEM41B-deficient T cells, we overexpressed CD5 in these cells (Fig. 6e, f). Notably, the enhanced response of TMEM41B-deficient T cells to antigen stimulation was partially reversed by CD5 overexpression (Fig. 6g), suggesting that CD5 downregulation contributes to the hyperresponsiveness of these cells.

To further explore the role of ER Ca^2+^ overload in CD5 downregulation and T cell hyperresponsiveness, we performed rescue experiments in TMEM41B-deficient T cells using the WT TMEM41B or its D91/93/94 A mutant. Re-expression of WT, but not the D91/93/94 A mutant of TMEM41B, largely restored CD5 expression and attenuated the hyperresponsiveness of TMEM41B-deficient T cells (Fig. 6h). These results indicate that ER Ca^2+^ overload, through certain mechanism(s), downregulates CD5 in TMEM41B-deficient T cells, thereby heightening their responsiveness to antigen stimulation.

### TMEM41B-deficient T cells exhibit heightened responses to infections

Finally, we assessed the physiological role of TMEM41B deficiency in T cells in vivo. As demonstrated earlier, TMEM41B-deficient naive T cells exhibited increased cell size and signs of metabolic activation, suggesting enhanced activity. To explore this further, we evaluated cell cycle entry by staining for Ki67, a marker of proliferating cells. A higher percentage of Ki67-positive TMEM41B-deficient T cells was observed compared to WT T cells (Supplementary Fig. S6a, b), suggesting that these cells had exited quiescence under steady-state conditions.

**Fig. 7:**
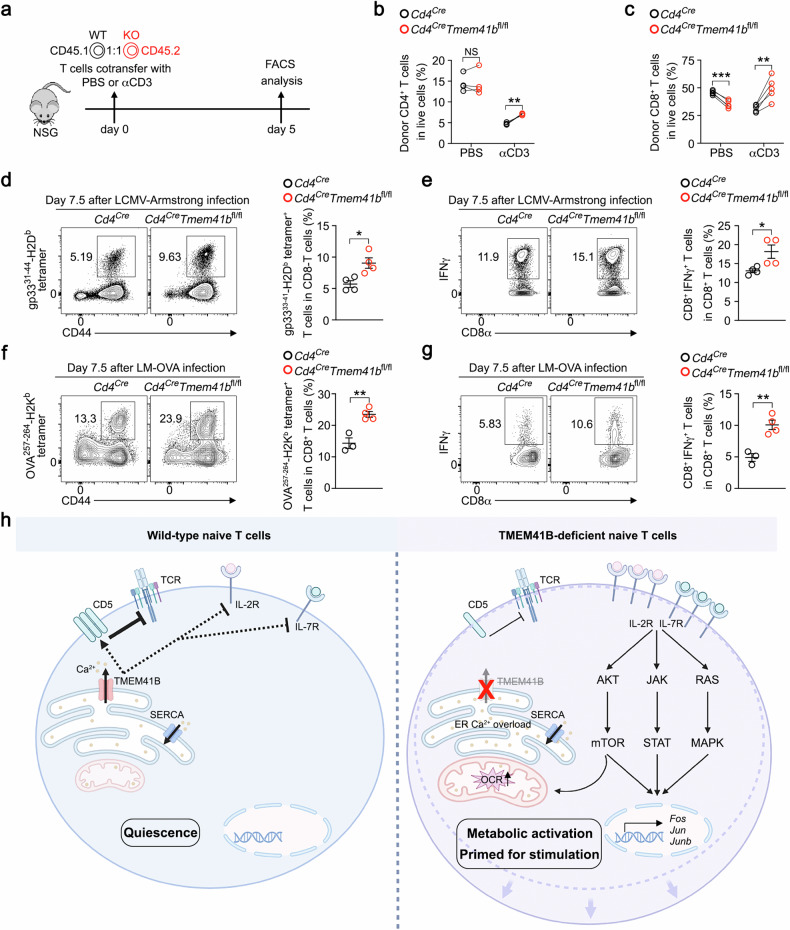
TMEM41B-deficient T cells exhibit increased responsiveness to infections. **a** Experimental design for the in vivo T cell deletion utilizing αCD3 antibody. **b** Percentages of CD4 T cells from control (*Cd4*^*Cre*^) or TMEM41B-deficient (*Cd4*^*Cre*^*Tmem41b*^*fl/fl*^) mice in NSG mice treated with either PBS or αCD3 antibody (*n* = 4 mice). **c** Percentages of CD8 T cells from control or TMEM41B-deficient mice in NSG mice treated with either PBS or αCD3 antibody (*n* = 5 mice). **d** Flow cytometry analysis of the percentages of H-2D^b^-gp33^+^ CD8 T cells in the spleens of control and TMEM41B-deficient mice 7.5 days after LCMV Armstrong infection. Representative plots and statistical analysis are shown (*n* = 4 mice). **e** Flow cytometry analysis of the percentages of IFNγ^+^ CD8 T cells (after ex vivo stimulation with gp33 peptide) in the spleens of control and TMEM41B-deficient mice 7.5 days after LCMV Armstrong infection. Representative plots and statistical analysis are shown (*n* = 4 mice). **f** Flow cytometry analysis of the percentages of H-2K^b^-OVA^+^ CD8 T cells in the spleens of control and TMEM41B-deficient mice 7.5 days after LM-OVA infection. Representative plots and statistical analysis are shown (*n* = 3 mice in control group, *n* = 4 mice in *Cd4*^*Cre*^*Tmem41b*^*fl/fl*^ group). **g** Flow cytometry analysis of the percentages of IFNγ^+^ CD8 T cells (after ex vivo stimulation with OVA^257–264^ peptide) in the spleens of control and TMEM41B-deficient mice 7.5 days after LM-OVA infection. Representative plots and statistical analysis are shown (*n* = 3 mice in control group, *n* = 4 mice in *Cd4*^*Cre*^*Tmem41b*^*fl/fl*^ group). **h** A working model of TMEM41B functioning as an ER Ca^2+^ release channel to maintain naive T cell quiescence and responsiveness. Data represent mean ± SEM; two-tailed paired *t*-test in **b** and **c**; unpaired *t*-test in **d**–**g**; *P < 0.05, **P < 0.01, *** *P* < 0.001, NS not significant.

Previous studies have shown that the loss of metabolic quiescence in naive T cells leads to resistance to activation-induced cell death, which has been used to represent the loss of peripheral tolerance^45,46^. We hypothesized that peripheral, TCR-induced deletional tolerance might be impaired in TMEM41B-deficient T cells. To test this, we co-transferred WT and TMEM41B-deficient naive T cells at a 1:1 ratio into T cell-deficient NSG mice and administered either αCD3 antibody or PBS as a control (Fig. 7a). TMEM41B-deficient T cells exhibited relative resistance to αCD3-induced T cell deletion (Fig. 7b, c), indicating reduced susceptibility to TCR-induced deletion in these cells. However, given that *Cd4*^*Cre*^*Tmem41b*^*fl/fl*^ mice did not manifest evident signs of autoimmunity by 6 months of age (data not shown), long-term monitoring is required to unveil spontaneous phenotypes.

Subsequently, we investigated the influence of TMEM41B on T cell response to infection. Although there was a slight reduction of naive CD8 T cells in *Cd4*^*Cre*^*Tmem41b*^*fl/fl*^ mice (Supplementary Fig. S2d), the percentages of antigen-specific CD8 T cells recognizing the lymphocytic choriomeningitis virus (LCMV) were significantly elevated in *Cd4*^*Cre*^*Tmem41b*^*fl/fl*^ mice compared to control mice during the peak response of LCMV Armstrong infection (Fig. 7d). Upon ex vivo restimulation with the gp33 peptide, there were more IFNγ^+^ cells in *Cd4*^*Cre*^*Tmem41b*^*fl/fl*^ mice than in control mice (Fig. 7e). Similar observations were made in a bacterial infection model with *Listeria monocytogenes* expressing the chicken ovalbumin (LM-OVA) (Fig. 7f, g). Thus, TMEM41B-deficient CD8 T cells displayed more robust responses in acute infections than control CD8 T cells, consistent with our in vitro findings that TMEM41B-deficient naive T cells are metabolically active and hyperreactive to antigen stimulation.

## Discussion

In this study, we demonstrate that TMEM41B functions as a concentration-dependent Ca^2+^ channel, releasing ER Ca^2+^ to prevent Ca^2+^ overload within the ER. ER Ca^2+^ overload drives TMEM41B-deficient T cells into a metabolically activated yet immunologically naive state, revealing a previously unappreciated but pivotal role of ER Ca^2+^ in maintaining metabolic quiescence and responsiveness of naive T cells, as depicted in Fig. 7h.

The SERCA family ATPases continuously pump Ca^2+^ from cytosol into ER lumen^6^, establishing a substantial electrochemical gradient of Ca^2+^ between the ER lumen and cytosol. In activated T cells and various other stimulated cell types, IP3R-mediated rapid release of ER Ca^2+^ store triggers SOCE, a process crucial for T cell activation^8,9^. In naive T cells and other resting/quiescent cells, ER also passively releases Ca^2+^^10–12^, yet Ca^2+^ channel(s) responsible for such steady-state ER Ca^2+^ release was elusive^13–15^. Our data establish that TMEM41B is a bona fide ER Ca^2+^ release channel. Supporting evidence includes: (1) TMEM41B deficiency resulting in ER Ca^2+^ overload (Fig. 1); (2) overexpression of TMEM41B depleting ER Ca^2+^ (Fig. 1); (3) overexpression of a plasma membrane-targeted TMEM41B inducing Ca^2+^ influx (Fig. 2); (4) Ca^2+^ channel activity of purified recombinant TMEM41B in an in vitro single-channel assay (Fig. 3); (5) TMEM41B mutant exhibiting altered Ca^2+^ channel activity (Fig. 3). Thus, TMEM41B is a long-sought ER Ca^2+^ release channel that contributes to the passive release of ER Ca^2+^ under steady state.

Given the vital roles of Ca^2+^ in physiology and the distinct concentrations of Ca^2+^ in various compartments, known Ca^2+^ channels are rigorously gated by diverse mechanisms, encompassing ligand binding, voltage sensitivity, mechanical force, and more. Our electrophysiological data demonstrate that the opening of TMEM41B depends on Ca^2+^ concentration, exhibiting increased opening at higher Ca^2+^ concentrations. This characteristic aligns with the physiological role of TMEM41B in preventing ER Ca^2+^ overload. When ER Ca^2+^ levels surpass a certain threshold, deviating from the established setpoint or homeostasis, TMEM41B is activated to release ER Ca^2+^, thus averting the accumulation of harmful levels of Ca^2+^ in the ER. Future structural studies will provide details of TMEM41B-mediated Ca^2+^ transport and the exact gating mechanism.

Biochemically, TMEM41B exhibits phospholipid scramblase activity^32,33^. Intriguingly, it is known that a single protein can serve dual functions as both an ion channel and a scramblase. For instance, the TMEM16 family of transmembrane proteins functions as both Ca^2+^-activated ion channels and phospholipid scramblases^47^. We speculate that TMEM41B, and potentially its counterparts TMEM41A, TMEM64, and VMP1 (TMEM49)^48^, may constitute another family of transmembrane proteins endowed with both ion channel and scramblase activities, warranting further research. In alignment with this notion, TMEM16 proteins have been shown to regulate SARS-CoV-2 infection by modulating Ca^2+^ oscillations in infected cells^49^. Notably, both TMEM41B and VMP1 are essential for the infection of various viruses, including SARS-CoV-2^29–31^. It has been recently shown that Ca^2+^ microdomains on the ER are essential for membrane budding^50^. Considering TMEM41B’s ability to release ER Ca^2+^ to cytosol and the reliance of viruses on host ER for vesicle budding, TMEM41B may control viral infection through its Ca^2+^ channel activity, which warrants future investigations.

Our previous study demonstrated the involvement of VMP1 in ER Ca^2+^ release in T cells^37^. Whether VMP1 is a Ca^2+^ channel remains to be determined. At cellular level, both TMEM41B- and VMP1-deficient cells manifest ER Ca^2+^ overload, and the overexpression of either protein depletes ER Ca^2+^, implying functional similarities between these two proteins. A noteworthy distinction is evident in VMP1-deficient T cells, where mitochondrial Ca^2+^ overload results in massive peripheral T cell death^37^. In contrast, TMEM41B-deficient T cells do not exhibit mitochondrial Ca^2+^ overload. It is possible that VMP1-deficient T cells experience more severe Ca^2+^ overload than TMEM41B-deficient cells, leading to the overflow of Ca^2+^ from the ER to mitochondria. Alternatively, VMP1 may localize at the ER–mitochondria junction, modulating ER–mitochondria Ca^2+^ transfer. Consequently, although both TMEM41B and VMP1 contribute to ER Ca^2+^ release, the distinct consequences of ER Ca^2+^ overload in TMEM41B- and VMP1-deficient T cells may hinge on the extent of Ca^2+^ overload in the ER or the manner in which ER Ca^2+^ is released, which warrants future investigations.

The metabolic phenotype exhibited by TMEM41B-deficient T cells is unique. Unlike the previously reported regulators of T cell quiescence, such as TSC1^51^, PTEN^52^ and Foxo1^53^, where metabolic activation was consistently accompanied by spontaneous T cell activation, TMEM41B-deficient T cells maintained an immunologically naive state while displaying significantly heightened metabolic activities. Marked increases in mitochondrial mass, mitochondrial membrane potential, OCR, ECAR, ROS levels, and cell size in TMEM41B-deficient naive T cells collectively point to metabolic activation, decoupled from immunological activation in these cells. Despite their immunological naivety, TMEM41B-deficient T cells exhibited features of “T cell activation”, including the downregulation of *Klf2* and upregulation of *Fos*, *Jun* and *Myc*, suggesting that these cells are in a transcriptionally primed state. Coupled with the downregulation of CD5, TMEM41B-deficient T cells are poised for antigen stimulation at both metabolic and signaling levels, explaining their heightened responsiveness to antigen stimulation, particularly at suboptimal antigen doses.

An important finding in this study is that the level of ER Ca^2+^ regulates the expression of key receptors on naive T cells, including IL-2 receptor α chain, β chain, common γ chain, IL-7Rα chain, and CD5. While the transcriptional changes that we observed suggest a direct regulatory effect at the mRNA level, other mechanisms may also contribute to the upregulation of IL-2/IL-7 receptors and the downregulation of CD5. ER Ca^2+^ homeostasis is closely linked to cellular stress responses, such as the unfolded protein response (UPR), which can activate transcription factors that influence the expression of various genes, including cytokine receptors. Additionally, post-transcriptional mechanisms, such as enhanced mRNA stability or increased protein translation, could also contribute to receptor upregulation. It is also possible that TMEM41B deficiency might alter receptor trafficking or degradation, resulting in higher surface expression of IL-2 and IL-7 receptors. The detailed mechanisms of such regulation await further investigation. Nevertheless, the increased expression of IL-2/IL-7 receptors and associated signaling events in TMEM41B-deficient T cells can only be reversed by WT TMEM41B but not a Ca^2+^ channel activity-defective mutant (D91/93/94 A), demonstrating that the metabolic activation of naive TMEM41B-deficient T cells is attributed to the Ca^2+^ channel activity rather than other functions of TMEM41B. Thus, ER Ca^2+^ plays a previously unappreciated role in maintaining the metabolic quiescence of resting naive T cells by suppressing aberrant signaling from IL-2 and IL-7. Conversely, the increased responsiveness of TMEM41B-deficient T cells implies that targeting TMEM41B or ER Ca^2+^ represents a novel strategy to amplify T cell response during infections.

Sustained Ca^2+^ entry through SOCE is essential for activating the NFAT pathway. The diminished SOCE in TMEM41B-deficient T cells may result in altered or reduced NFAT activity. CD25, the α chain of the IL-2 receptor, is closely tied to sustained Ca^2+^ signaling. Consequently, while TMEM41B-deficient T cells more robustly upregulate activation markers like CD44 and CD69 compared to control cells, reduced SOCE may limit their ability to upregulate CD25 at higher doses of CD3 stimulation (Fig. 6c, d). This highlights the complexity of TMEM41B’s role in T cells: on one hand, the upregulation of IL-2/IL-7 receptors and downregulation of CD5 render TMEM41B-deficient T cells more responsive to TCR stimulation; on the other hand, reduced SOCE may dampen the NFAT pathway, which is crucial for full activation of T cells.

TMEM41B exhibits broad expression across various tissues. Although not specifically investigated in this study, it is plausible that TMEM41B-mediated ER Ca^2+^ release also plays a role in regulating the homeostasis of other immune cells. Beyond its impact on immune system, dysregulations of ER Ca^2+^ are implicated in a myriad of human diseases^2,4^. The loss of TMEM41B causes spinal muscular atrophy (a neurodegenerative disease) in worms and mice^54,55^. Additionally, deletion of TMEM41B in the liver induces nonalcoholic hepatosteatosis in mice^32^. In humans, single nucleotide polymorphisms in TMEM41B are associated with viral infections such as SARS-CoV-2^29^. Targeting TMEM41B-mediated ER Ca^2+^ release holds promise for therapeutic interventions in these diverse pathological conditions.

## Materials and methods

### Animals

C57BL/6 mice (Cat# 000664, RRID: IMSR_JAX: 000664), *Cd4*^*Cre*^ mice (Cat# 022071, RRID: IMSR_JAX: 022071), Cas9 transgenic mice (Cat# 026430, RRID: IMSR_JAX: 026430), NSG mice (Cat# 005557, RRID: IMSR_JAX: 005557) and *Rptor* flox mice (Cat# 013188, RRID: IMSR_JAX: 013188) originally came from The Jackson Laboratory. *Tmem41b* flox mice (exons 3–5) were generated by gene targeting service provided by Cyagen Biosciences. Genotyping primers used were as follows: *Tmem41b*-F1: CTGTGTGAGACTAAAACCAGTGAG; *Tmem41b-*R1: CAGAAACACATCCTAGGTCAGATGC; *Tmem41b-*R2: AAATGATGCTGACCACTTTCAGGG; *Rptor*-F: CTCAGTAGTGGTATGTGCTCAG; *Rptor-*R: GGGTACAGTATGTCAGCACAG. *Cd4*^*Cre*^ mice, *Tmem41b* flox mice and *Rptor* flox mice were C57BL/6 J background. *Vmp1* flox mice have been reported previously^37^. Age and sex-matched littermates were used as control in all experiments. Mice were housed under specific pathogen-free conditions at the Laboratory Animal Research Center of Tsinghua University (Beijing, China). The facility was approved by Beijing Administration Office of Laboratory Animal. All animal works were approved by Institutional Animal Care and Use Committee (IACUC).

### Cell lines

HEK293T cells (Cat# CRL-3216, RRID: CVCL_0063) were obtained from ATCC. Plat-E cells were obtained from Hai Qi’s laboratory at Tsinghua University. HEK293T-G-CEPIA1er-sensor cells, HEK293T-STIM1-KO cells, and HEK293T-ORAI1-KO cells were generated in our laboratory. Further details can be found in our previous studies^37^. All cell lines were cultured in DMEM (Gibco, Cat# C1195500) supplemented with 10% fetal bovine serum (FBS) (Gemini), 2 mM glutamine (Macgene, Cat# CC009), 100 units/mL of penicillin and 100 μg/mL of streptomycin (Macgene, Cat# CC004) at 37 °C in a humidified incubator containing 5% CO_2_. All cell lines were tested for mycoplasma by the TransDect^TM^ PCR Mycoplasma detection Kit (TRAN, Cat# FM311) and were confirmed to be negative.

### Primary T cell culture

Primary T cells isolated from spleen and lymph nodes were activated overnight with 1 μg/mL anti-CD3 (BioXCell, BP0001-1, RRID: AB 1107634) and 1 μg/mL anti-CD28 (BioXCell, BE0015-1, RRID: AB_1107624). Cells were passaged every 1–2 days at a density of 1–2 × 10^6^ cells/mL with 2 ng/mL IL-2 (PeproTech, Cat# 200-02-1000). T cells were cultured in T cell medium (TCM) composed of RPMI 1640 medium (Gibco, Cat# C1187550) supplemented with 5% FBS, 2 mM glutamine, 55 μM β-mercaptoethanol (Solarbio, Cat# M8210), 1 mM sodium pyruvate (Macgene, Cat# CC007), 100 units/mL penicillin, 100 μg/mL streptomycin and 2 ng/mL IL-2.

### Retrovirus production and viral transduction

Retroviruses were generated by transfecting Plat-E cells with associated plasmids using Chemifect^TM^ (Fengrui, Cat# FR-01) according to the manufacturer’s protocol. The viral supernatant was harvested at 48 h and 72 h post transfection, filtered via 0.45-μm filters, aliquoted, and stored at –80 °C. Primary T cells were activated with 1 μg/mL αCD3 and αCD28, and suspended in 1 mL TCM in 12-well plate. Subsequently, 2 mL of retrovirus and 8 μg/mL polybrene (Sigma-Aldrich, Cat# H9268) were added into T cells 20–24 h post activation, followed by 2000× *g* centrifugation at 33 °C for 2 h. The plate was then returned to the incubator. After 6 h of incubation, the cells were transferred to 10-cm dish with fresh medium containing 2 ng/mL IL-2.

### CRISPR knockout of individual gene in HEK293T cells

Single guide RNAs (sgRNAs) were cloned into LentiCRISPRv2 (Addgene, Cat# 52961) to knock out specific genes in HEK293T cells. Monoclonal knockout cell lines were generated by transiently transfecting HEK293T cells with LentiCRISPRv2 plasmids targeting specific genes using Chemifect^TM^ following the manufacturer’s protocol. The transfected cells were then selected with 3 μg/mL puromycin (Invitrogen, Cat# 1566-1) for 72 h. Subsequently, the cells were plated in 96-well plates at approximately one cell per well in DMEM supplemented with 15% FBS. After 10 days, single clones were expanded and verified by immunoblotting. The target sequences of the sgRNAs were as follows: sgNon-targeting: TTCGCACGATTGCACCTTGG; sg*TMEM41B*: AGGCACCAAGTCCAGAACAC.

### Ca^2+^ measurement with Fluo4/Fura Red by flow cytometry

Fura Red (Invitrogen, Cat# F3021) and Fluo4 (Invitrogen, Cat# F14217) were used for Ca^2+^ measurement following a published protocol^37^. In all Ca^2+^ assays, HEK293T cells were plated at the same density in 6-well plates one day before the experiment to exclude the influence of cell density. 1 × 10^6^ control or gene-modified HEK293T cells or primary T cells were resuspended in 250 μL Ca^2+^ assay medium (DMEM for HEK293T cells, RPMI 1640 for primary T cells, both supplemented with 25 mM HEPES (pH 7.4) and 2.5% FBS). Fluo4 and Fura Red were mixed together and prepared as 2× solution in 250 μL Ca^2+^ assay medium. Then, the 250 μL medium containing cells and the 250 μL medium with 2× Fluo4 and Fura Red mixture were mixed together, resulting in a final concentration of 2 μM for both Fura Red and Fluo4. The mixtures were then incubated at 37 °C for 30 min in the dark. Cells were washed once with Ca^2+^ assay medium and resuspended in 800 μL Ca^2+^ assay medium for flow cytometry analysis.

For the measurement of ER Ca^2+^ and SOCE, the dye-loaded cell suspension was supplemented with a final concentration of 2 mM EGTA (Amresco, Cat# 0732) before being placed on cytometer. Given that the concentration of Ca^2+^ is 0.42 mM in RPMI 1640 and 1.8 mM in DMEM, 2 mM EGTA can completely chelate the extracellular Ca^2+^. We adjusted the baseline intensities for Fluo4 (FITC channel) and Fura Red (PerCP-Cy5.5 channel), and recorded for 60 s. Then we removed the tube from the cytometer, and quickly added TG (Cell Signaling Technology, Cat# 12758S) to a final concentration of 1 μM. Samples were quickly mixed, returned to the cytometer, and recorded for another 420 s. Then, without interrupting the acquisition/recording, we removed the tube from the cytometer, and quickly added CaCl_2_ to a final concentration of 2 mM (excluding the Ca^2+^ in assay medium). Samples were quickly mixed, returned to the cytometer and recorded for another 420 s. All measurements were performed at 25 °C.

For the measurement of Ca^2+^ influx in cells transfected with plasma membrane-targeted TMEM41B (TMEM41B-K4A), dye-loaded cells were recorded for 60 s. Subsequently, without interrupting the acquisition/recording, we removed the tube from the cytometer, swiftly added 8 mM CaCl_2_ (final concentration), and promptly mixed the samples. The tube was then returned to the cytometer for an additional recording of 240 s.

For the measurement of ER Ca^2+^ store in WT and TMEM41B-deficient T cells, freshly isolated T cells were loaded with Fluo4 (FITC channel) and Fura Red (PerCP-Cy5.5 channel). Then, the cell suspension was supplemented with a final concentration of 2 mM EGTA before being placed on the cytometer and recorded for 60 s. Subsequently, the tube was removed from the cytometer (without stopping the acquisition/recording), and 1 μM Ca^2+^ ionophore ionomycin (Biovision, Cat# 1566-1) (final concentration) was quickly added to the samples. After quick mixing, the samples were returned to the cytometer and recorded for another 240 s.

For the measurement of TG-induced Ca^2+^ influx (SOCE) in WT and TMEM41B-deficient T cells, freshly isolated T cells were loaded with Fluo4 (FITC channel) and Fura Red (PerCP-Cy5.5 channel). Then, the dye-loaded cell suspension was recorded for 60 s. Subsequently, without interrupting the acquisition/recording, we removed the tube from the cytometer, swiftly added 20 nM TG (final concentration), and promptly mixed the samples. The tube was then returned to the cytometer for an additional recording of 240 s.

For the measurement of TCR-induced Ca^2+^ influx in WT and TMEM41B-deficient T cells, dye-loaded T cells were incubated with 10 μg/mL αCD3-biotin (BioLegend, Cat# 100304), followed by the addition of 10 μg/mL purified streptavidin (BioLegend, Cat# 280302) for cross-linking. SOCE was examined by flow cytometry as described above.

### Ca^2+^ measurement using Ca^2+^ sensor G-CEPIA1er by flow cytometry

HEK293T cells stably expressing G-CEPIA1er sensor were established by lentiviral transduction^37^. In overexpression studies, sensor cells were transfected with the indicated constructs with a BFP reporter, and the influence of overexpression on the sensor intensity was measured on BFP^high^ cells. For the fluorescence measurement of G-CEPIA1er, 1 × 10^6^ cells were suspended in 800 μL Ca^2+^ assay medium. The cell suspension was placed on the cytometer, and G-CEPIA1er (GFP channel) fluorescence was recorded for 60 s. Subsequently, the tube was removed from cytometer, and TG was quickly added to achieve a final concentration of 1 μM. Samples were quickly mixed, returned to the cytometer, and fluorescence was recorded for another 840 s. The total assay time for each sample was 900 s. The voltages for GFP channel (for G-CEPIA1er) were kept unchanged for all samples.

### Overexpression experiments

To overexpress proteins in HEK293T cells, the full-length cDNAs of human TMEM41B or its mutant TMEM41B-K4A were cloned into a small vector pCMV with a backbone of 3.7 kb. This vector lacks a fluorescent marker; therefore, a pCMV-BFP construct was co-transfected to facilitate gating out of BFP-high cells for analysis via flow cytometry.

To overexpress proteins in T cells, the full-length cDNAs of mouse TMEM41B, its mutant TMEM41B-D91/93/94 A, or CD5 were cloned into pMIG vector with Thy1.1 as a reporter marker. Retrovirus production, T cell activation and spin infection were performed as described above. At 72 h post spin infection, Thy1.1-positive T cells were gated out for analysis via flow cytometry.

### Immunofluorescence

HEK293T cells were seeded onto poly-D-lysine Cellware 12-mm coverslips (Corning, Cat# 354086). On the next day, cells were transfected with the indicated plasmids. After 24 h of transfection, cells were rinsed once with PBS and stained with WGA (Invitrogen, Cat# W32466) for 10 min at 37 °C. Then cells were washed three times with PBS and fixed with 4% para-formaldehyde (Beyotime, Cat# P0099) in PBS for 15 min at room temperature. Subsequently, cells were washed twice with PBS and permeabilized with pre-chilled methanol (–20 °C) for 10 min on ice. After three washes with PBS, the coverslips were blocked with 5% BSA for 30 min at room temperature. Following blocking, cells were incubated with anti-FLAG primary antibody (Sigma-Aldrich, Cat# F1804) (diluted 1:500 in 5% BSA) for 1–3 h at room temperature. Then, coverslips were washed four times with PBS and incubated with goat anti-mouse Alexa Fluor Plus 488 secondary antibody (Thermo Fisher Scientific, Cat# A32723) (diluted 1:1000 in 5% BSA) for 1 h at room temperature in the dark. Finally, coverslips were washed four times with PBS and mounted onto glass using ProLong^TM^ Gold Antifade Mountant (Thermo Fisher Scientific, Cat# P36934) with DAPI (BioLegend, Cat# 422801), and imaged using a Zeiss780 confocal microscope with a 63× oil lens.

### Purification of recombinant TMEM41B proteins

To purify recombinant TMEM41B proteins, 8 × 10^6^ HEK293T cells were seeded in a 15-cm plate 16 h before transfection. Transfection was performed with 20 μg of plasmids encoding FLAG-TMEM41B or FLAG-TMEM41B-D91/93/94 A using Chemifect^TM^ according to the manufacturer’s protocol. After 36 h of transfection, cells were harvested, washed twice with ice-cold PBS, and lysed with 1% DDM lysis buffer (containing 20 mM Tris-Cl, pH 8.0, 2 mM CaCl_2_, 200 mM NaCl, 1% DDM, 0.2% (w/v) CHS, 0.012% (w/v) glyco-diosgenin (GDN) (Anatrace, Cat# GDN101), and 1× EDTA-free protease inhibitor cocktail (Yeasen, Cat# 20123ES50) at a volume approximately five times that of the volume of the cell pellet for cell lysis. Lysis was performed for 2 h with gentle rotation at 4 °C. The lysate was centrifuged at 15,000 rpm for 30 min at 4 °C, and the supernatant was incubated with anti-DYKDDDDK G1 Affinity resin (GenScript, Cat# L00907) for 3 h with gentle rotation at 4 °C. The beads were washed five times with wash buffer (containing 20 mM Tris-Cl, pH 8.0, 200 mM NaCl, 2 mM CaCl_2_, and 0.008% GDN). Bound proteins were eluted with 500 μL wash buffer containing 0.125 mg/mL FLAG peptide (GenScript, Cat# RP10586) for 2 h with gentle rotation at 4 °C. The buffer containing the eluted protein was filtered through a 0.22-μm spin filter and directly loaded onto an Enrich SEC650 column (Bio-Rad) for size exclusion chromatography (SEC). Fractions containing the proteins were collected and analyzed.

### Western blot

After treatment as indicated, cells were collected and washed with cold PBS. Subsequently, cells were lysed with lysis buffer containing 1% Triton X-100, 40 mM HEPES, pH 7.4, 10 mM β-glycerol phosphate, and 10 mM pyrophosphate, supplemented with EDTA-free protease inhibitor cocktail on ice for 15 min. The soluble fractions of cell lysates were isolated by centrifugation at 15,000 rpm for 10 min at 4 °C. Proteins were denatured by the addition of 6× SDS sample buffer and boiling for 10 min at 95 °C. Finally, the samples were subjected to SDS-PAGE (LABLEAD, Cat# P41215), native gel electrophoresis, and immunoblotting analysis. The following antibodies were used: rabbit polyclonal anti-TMEM41B (Invitrogen, Cat# PA5-53259), mouse monoclonal anti-β-actin (Santa Cruz, Cat# sc-47778), rabbit monoclonal anti-phospho-eIF2α-Ser51 (clone D9G8) (Cell Signaling Technology, Cat# 3398), rabbit monoclonal anti-eIF2α (clone D7D3) (Cell Signaling Technology, Cat# 5324), recombinant anti-αCOPI/COPA (clone EPR14273[B]) (Abcam, Cat# ab181224), rabbit polyclonal anti-βCOP (Abcam, Cat# ab2899) and mouse monoclonal anti-FLAG (DYKDDDDK) (Sigma-Aldrich, Cat# F1804).

### Electrophysiology recording of TMEM41B by planar lipid bilayers

The electrophysiological experiment was performed with the Planar Lipid Bilayer Work station (BLM Workstation). Chamber and Cup separate the solution into two compartments that were connected with a 200-μm hole. We define Cup as the *cis* side, and Chamber as the *trans* side. Different recording solutions were added in *cis* and *trans* sides, respectively. Bath solutions were listed as (1) *cis*: 500 mM KCl, 5 mM HEPES, pH 6.5; *trans*: 50 mM KCl, 5 mM HEPES, pH 6.5. (2) *cis*: 150 mM CaCl_2_, 5 mM HEPES, pH 6.5; *trans*: 15 mM CaCl_2_, 5 mM HEPES, pH 6.5. (3) *cis*: 500 mM KCl, 10 mM CaCl_2_, 5 mM HEPES, pH 6.5; *trans*: 50 mM KCl, 100 mM CaCl_2_, 5 mM HEPES, pH 6.5. The phospholipids (phosphatidylcholine:phosphatidylserine = 3:2) dissolved in decane were painted softly into the small hole in Cup to form lipid bilayer. All lipids were bought from Avanti (Avanti Polar Lipids, USA). Purified TMEM41B or mutant protein was added in *cis* side to incorporate into planar lipid bilayer. The duration of each single-channel recording was longer than 2 min. Single-channel currents were recorded under voltage-clamp mode using a Warner bilayer clamp amplifier BC-535 (Warner Instruments, USA), and filtered at 1–2 kHz. The recording frequency was 10 kHz. Analog voltage was digitized with Digidata 1440 A (Molecular Devices, USA), and the data was stored by pCLAMP 10.4. The amplitude of events and open probability (*P*_open_) were detected by Clampfit. Events with opening time less than 1.5 ms were ignored. Single-channel conductance was determined by fitting to Gaussian function equations. The equilibrium potential was calculated using the Nernst equation and Goldman–Hodgkin–Katz flux equation.

$${\rm{Open}}\; {\rm{probability}}:\,{P}_{{open}}=\frac{{t}_{{open}}}{T}$$

$${\rm{For}}\; {\rm{monovalent}}\; {\rm{ion}}:\,{E}_{{rev}}=\frac{{RT}}{F}\mathrm{ln}\frac{{P}_{X+}{[{X}^{+}]}_{{out}}+{P}_{Y+}{[{Y}^{+}]}_{{out}}}{{P}_{X+}{[{X}^{+}]}_{{in}}+{P}_{Y+}{[{Y}^{+}]}_{{in}}}$$

$${\rm{For}}\; {\rm{divalent}}\; {\rm{ion}}:\,{E}_{{rev}}=\frac{{RT}}{F}{\mathrm{ln}}\frac{{P}_{X+}{\left[{X}^{+}\right]}_{{out}}(1+{e}^{\frac{F* {E}_{{rev}}}{{RT}}})+4{P}_{Y2+}{[{Y}^{2+}]}_{{out}}}{{P}_{X+}{[{X}^{+}]}_{{in}}(1+{e}^{\frac{F* {E}_{{rev}}}{{RT}}})+{4P}_{Y2+}{[{Y}^{2+}]}_{{in}}{e}^{\frac{F* {Erev}}{{RT}}}}$$

### Flow cytometry

Singe cell suspensions were prepared from thymi, spleens, and lymph nodes by grinding through 70-μm strainer. Erythrocytes were depleted by hypotonic lysis. For staining of surface markers, cells were incubated in FACS buffer (PBS supplemented with 1% calf serum, 1% penicillin/streptomycin and 2 mM EDTA) with the indicated combinations of antibodies for 15 min at 4 °C, together with Fc blockade (2G4) to prevent non-specific binding. Afterward, cells were washed twice with FACS buffer, and DAPI (BioLegend, Cat# 422801) staining was used to exclude dead cells. Samples were recorded using an LSR Fortessa cytometer (BD) and analyzed with FlowJo software (BD). The following antibodies were used: CD4(GK1.5)-APC (BioLegend, Cat# 100412), CD8α(53-6.7)-eFluor 450 (eBioscience, Cat# 48-0081-82), CD44(IM7)-PerCP/Cyanine5.5 (BioLegend, Cat# 103032), CD62L(MEL-14)-PE-Cyanine7 (eBioscience, Cat# 25-0621-82), CD69(H1.2F3)-PE (BioLegend, Cat# 104508), CD25(PC61)-FITC (BioLegend, Cat# 102006), CD122(TM-b1)-PE-Cyanine7 (eBioscience, Cat# 25-1222-82), CD132(TUGm2)-PE (BioLegend, Cat# 132306), CD127(A7R34)-PE (BioLegend, Cat# 135010) and CD5(53-7.3)-PE (BioLegend, Cat# 100608); FITC Rat IgG1, λ Isotype Ctrl Antibody (BioLegend, Cat# 401914) for CD25(PC61)-FITC; PE/Cyanine7 Rat IgG2β, κ Isotype Ctrl Antibody (BioLegend, Cat# 400617) for CD122(TM-b1)-PE-Cyanine7, PE Rat IgG2β, κ Isotype Ctrl Antibody (BioLegend, Cat# 400607) for CD132(TUGm2)-PE, PE Rat IgG2α, κ Isotype Ctrl Antibody (BioLegend, Cat# 400508) for CD127(A7R34)-PE and CD5(53-7.3)-PE.

For apoptosis assays, cells were incubated with CD4(GK1.5)-APC (BioLegend, Cat# 100412), CD8α (53-6.7)-PE (BioLegend, Cat# 100708) and Fc blockade at 4 °C for 15 min. After incubation, cells were washed twice with binding buffer in Annexin V-FITC Apop Kit (Invitrogen, Cat# BMS500FI-300). Subsequently, cells were stained with Annexin V following the instructions of Annexin V-FITC Apop Kit. DAPI staining was used to exclude dead cells.

For measurement of mitochondrial Ca^2+^, cells were washed once with serum-free RPMI 1640 medium with 5 mM EGTA and then incubated with 1 μM Rhod-2-AM (Invitrogen, Cat# R1244) together with antibodies for CD4(GK1.5)-APC (BioLegend, Cat# 100412), CD8α(53-6.7)-eFluor 450 (eBioscience, Cat# 48-0081-82) and Fc blockade at 37 °C for 30 min. After incubation, cells were washed twice and resuspended in serum-free RPMI 1640 medium. DAPI staining was used to exclude dead cells.

For measurement of mitochondrial mass, cells were washed once with serum-free RPMI 1640 medium and then incubated with 200 nM MitoTracker® Green (Invitrogen, Cat# M7514) together with antibodies for CD4(GK1.5)-APC (BioLegend, Cat# 100412), CD8α(53-6.7)-eFluor 450 (eBioscience, Cat# 48-0081-82) and Fc blockade at 37 °C for 30 min. After incubation, cells were washed twice and resuspended in serum-free RPMI 1640 medium. DAPI staining was used to exclude dead cells.

For measurement of mitochondrial membrane potential, cells were incubated with 2 μM TMRM dye (Tetramethylrhodamine, methyl ester) (MCE, Cat# HY-D0984A) in RPMI 1640 medium at 37 °C for 1 h. Then cells were washed twice to remove TMRM dye and stained with CD4(GK1.5)-APC (BioLegend, Cat# 100412), CD8α (53-6.7)-eFluor 450 (eBioscience, Cat# 48-0081-82) and Fc blockade for 10 min at room temperature. DAPI staining was used to exclude dead cells.

For measurement of total ROS, cells were incubated with CD4(GK1.5)-APC (BioLegend, Cat# 100412), CD8α(53-6.7)-PE (BioLegend, Cat# 100708) and Fc blockade at 4 °C for 15 min. After being washed twice with pre-warmed HBSS, cells were incubated with 0 μM or 2 μM DCF (Invitrogen, Cat# 88-5930) at 37 °C for 10 min. Subsequently, cells were washed twice and further incubated in pre-warmed RPMI 1640 medium containing 10% FBS at 37 °C for 30 min. DAPI staining was used to exclude dead cells.

For phospho-flow assays, cells were first stained with surface markers together with LIVE/DEAD Fixable Near-IR (Thermo Fisher Scientific, Cat# L34976) and Fc blockade at 4 °C for 15 min. Cells were washed twice with FACS buffer. Permeabilization and staining of phosphorylated proteins were performed with Transcription Factor Staining Buffer kit (BD Pharmingen, Cat# 562574) according to the manufacturer’s instructions. The following antibodies were used: CD4(GK1.5)-APC (BioLegend, Cat# 100412), CD4(GK1.5)-PE (BioLegend, Cat# MA1-10220), CD8β(H35-17.2)-FITC (eBioscience, Cat# 11-0083-85), Phospho-Akt (Thr308) (D25E6) XP® Rabbit mAb (Cell Signaling Technology, Cat# 13038), Phospho-Akt (Ser473) (D9E) XP® Rabbit mAb (Cell Signaling Technology, Cat# 4060), Phospho-p70 S6 Kinase (Thr389) (Cell Signaling Technology, Cat# 9205), Phospho-S6 Ribosomal Protein (Ser235/236) (D57.2.2E) XP® Rabbit mAb (Alexa Fluor® 647 Conjugate) (Cell Signaling Technology, Cat# 4851), Phospho-STAT5 (Tyr694) Monoclonal Antibody (SRBCZX)-PE (Thermo Fisher Scientific, Cat# 12-9010-42), Phospho-ERK(E-4)-PE (Santa Cruz, Cat# sc-7383), Goat anti-Rabbit IgG (H + L) Highly Cross-Adsorbed Secondary Antibody (Alexa Fluor® 647 Conjugate) (Thermo Fisher Scientific, Cat# A21245), and Goat anti-Rabbit IgG (H + L) Highly Cross-Adsorbed Secondary Antibody (Alexa Fluor® 488 Conjugate) (Thermo Fisher Scientific, Cat# A32731).

### scRNA-seq and analysis

Lymphocytes from TMEM41B-deficient mice and WT littermates were stained with markers for TCRβ, CD44, CD62L, and CD25. TCRβ was used to identify both CD4 and CD8 T cells, and CD44 and CD62L were used to isolate naive T cells (CD44^–^CD62L^+^), while CD25 was used to exclude regulatory T cells. The TCRβ^+^CD44^–^CD62L^+^CD25^–^ naive T cells (sorted as the 20% lowest CD44^–^) from WT or TMEM41B-deficient mice were sorted for scRNA-seq analysis.

Then naive T cells from WT or TMEM41B-deficient mice were directly loaded onto a microfluidic chip, and libraries were prepared using the Singleron GEXSCOPETM Single Cell RNA-seq Kit (Singleron Biotechnologies), following the manufacturer’s protocol. Sequencing was performed on an Illumina NovaSeq 6000 platform, targeting ~13,000 cells per sample. Data alignment, filtering, and quantification were processed with CeleScope (v1.9.0), and subsequent analyzes were conducted using Seurat (v4.3.0). Cell-level quality control was performed by filtering cells based on the following criteria: (1) gene numbers between 500 and 5000, (2) total UMI counts between 1000 and 20,000, and (3) mitochondrial gene percentage less than 10%. After filtering, the top 2000 variable genes were selected for principal component analysis. The first 1–10 principal components, as determined by the JackStraw method, were used for UMAP and clustering analysis. Cluster-specific genes were identified using the FindAllMarkers function with a log_2_fold-change threshold of 0.25. Data visualization was achieved using FeaturePlot and VlnPlot functions. Signature gene set scoring was performed using the AddModuleScore function from Seurat, applied to selected gene sets.

UMAP is a dimensionality reduction technique used in scRNA-seq analysis to visualize cellular heterogeneity. In the UMAP plot presented in Fig. 4c, each dot represents an individual cell. This plot shows the cluster distribution of FACS-sorted naive (TCRβ^+^CD44^–^CD62L^+^CD25^–^) T cells from both WT and TMEM41B-deficient mice, where red dots represent knockout T cells, and green dots represent WT T cells. Additionally, the UMAP plot in Supplementary Fig. S3c displays the expression levels of key genes, including *Cd4*, *Cd8*, *Cd44*, and *Sell* (CD62L), to validate the sorting efficiency. The depth of color corresponds to the level of gene expression.

Cells with similar gene expression profiles cluster together, which reveals distinct cell populations or subtypes. Based on differences in transcriptional profiles, both WT and TMEM41B-deficient naive T cells were clustered into 13 distinct groups (clusters 0–12) (Supplementary Fig. S3d, e), with each cluster represented by a different color (Supplementary Fig. S3e). The heatmap in Supplementary Fig. S3d highlights the defining markers for each cluster. Supplementary Fig. S3f illustrates the percentage of control and TMEM41B-deficient naive T cells within each cluster. Additionally, we performed UMAP analyses separately for control (Fig. 4d) and TMEM41B-deficient naive T cells (Fig. 4e), which more directly shows the differences between these two groups.

GSEA is a computational method used to identify whether predefined gene sets show statistically significant and coordinated differences in expression across various cell types, clusters, or conditions within a dataset. In Fig. 4g, GSEA performed using the ssGSEA method revealed upregulated (red box) and downregulated (green box) gene sets in TMEM41B-deficient naive T cells compared to control naive T cells. Additionally, Supplementary Fig. S3i highlights upregulated gene sets related to amino acid metabolism in TMEM41B-deficient naive T cells.

SingleSeqGSet (GSEA in scRNA-seq) analysis examines whether the expression levels of genes in a particular gene set are consistently upregulated or downregulated in a specific group of cells, compared to the control. It highlights pathways or processes that are overrepresented in specific cell types, clusters, or conditions. Supplementary Fig. S3h illustrates the SingleSeqGSet analysis across all clusters of pooled naive T cells from both control and TMEM41B-deficient mice. The results show that clusters 0 and 1 are enriched in pathways associated with higher respiratory activity.

Violin plots are a type of data visualization that merge the features of both box plots and density plots, allowing for a detailed comparison of gene expression levels across different cell types or clusters. In Fig. 4f, h and Supplementary Fig. S3g, the violin plots illustrate the distribution of gene expression levels for representative genes in both control and TMEM41B-deficient naive T cells.

### Seahorse experiments

OCR and ECAR were measured with an XF96 extracellular flux analyzer (Agilent). Naive CD4 T cells were purified with MojoSort™ Mouse CD4 Naive T Cell Isolation Kit (BioLegend, Cat# 480040) according to the manufacturer’s instructions. Freshly isolated naive T cells were seeded on XF96 microplates (150,000 cells/well) that had been pre-coated with Cell-Tak^TM^ Cell and tissue adhesive according to the manufacturer’s instructions (Corning, Cat# 354240). The Seahorse XF Mito stress test kit (Agilent, Cat# 103015-100) was used to test OCR under different conditions. Firstly, cells were incubated in the mito stress test medium without any drugs, and four baseline recordings were assessed. Then maximal OCR was obtained by sequential injection of 2 μM oligomycin and 1 μM FCCP that uncoupled oxygen consumption from ATP. Finally, 1 μM rotenone/antimycin-A that inhibits complex I and III was injected. The Seahorse XF Glycolysis stress test kit (Agilent, Cat# 103020-100) was used to test ECAR under different conditions. Initially, cells were incubated in the glycolysis stress test medium without glucose, and four basal ECAR recordings were assessed. After sequential injection of 10 mM glucose and 4 μM oligomycin that inhibits mitochondrial ATP production, the energy production shifted to glycolysis. The increased ECAR revealed the maximum glycolytic capacity of T cells. Finally, 50 mM 2-DG was injected to inhibit glycolysis.

### Quantitative PCR (qPCR) analysis

Naive T cells (TCRβ^+^CD44^–^CD62L^+^CD25^–^) from WT and TMEM41B-deficient mice were sorted for qPCR analysis. Total RNA from sorted naive T cells was extracted using the RNA Easy Fast animal tissues/cells total RNA extraction kit (TIANGEN, Cat# DP451) following the manufacturer’s protocol. cDNA library was prepared by reverse transcription using FastKing one-step RT-PCR kit (TIANGEN, Cat# KR123) and further amplified by Taq SYBR® Green qPCR premix (LABLEAD, Cat# R0202) at CFX96 Real-Time PCR System (Bio-Rad). qPCR primers used in this study were: *Cd25*-Forward sequence: GCGTTGCTTAGGAAACTCCTGG; *Cd25*-Reverse sequence: GCATAGACTGTGTTGGCTTCTGC; *Cd132*-Forward sequence: GGAGCAACAGAGATCGAAGCTG; *Cd132*-Reverse sequence: CCACAGATTGGGTTATAGCGGC; *Cd127*-Forward sequence: CACAGCCAGTTGGAAGTGGATG; *Cd127*-Reverse sequence: GGCATTTCACTCGTAAAAGAGCC; *Jun*-Forward sequence: CAGTCCAGCAATGGGCACATCA; *Jun*-Reverse sequence: GGAAGCGTGTTCTGGCTATGCA; *Junb*-Forward sequence: GACCTGCACAAGATGAACCACG; *Junb*-Reverse sequence: ACTGCTGAGGTTGGTGTAGACG; *Myc*-Forward sequence: TCGCTGCTGTCCTCCGAGTCC; *Myc*-Reverse sequence: GGTTTGCCTCTTCTCCACAGAC; *Hprt*-Forward sequence: CTGGTGAAAAGGACCTCTCGAAG; *Hprt*-Reverse sequence: CCAGTTTCACTAATGACACAAACG.

### αCD3-induced T cell deletion in vivo

Naive CD4 or CD8 T cells were purified using FACS. WT (CD45.1) and TMEM41B-deficient T cells (CD45.2) were mixed at a 1:1 ratio (a total of 1 × 10^6^ cells) and co-transferred into NSG mice via tail vein. Subsequently, mice were injected with either 10 mg of anti-CD3ε antibody or PBS as a control. On day 5 post transfer, mice were sacrificed, and donor T cells in spleen were analyzed by flow cytometry. Splenocytes were stained with CD4(GK1.5)-APC (BioLegend, Cat# 100412) or CD8α(53-6.7)-APC (BioLegend, Cat# 100712), CD45.1(A20)-FITC (BioLegend, Cat# 110706), CD45.2(104)-PE (eBioscience, Cat# 12-0454-83), in the presence of Fc block in FACS buffer for 15 min at 4 °C. Cells were washed twice with FACS buffer and analyzed by an LSR Fortessa cytometer (BD). Dead cells were excluded by DAPI staining.

### LCMV Armstrong infection

The LCMV Armstrong strain was generously provided by Yuncai Liu’s lab at Tsinghua University. Mice were infected with LCMV Armstrong virus (1 × 10^5^ plaque-forming units) by intraperitoneal injection. On day 7.5 post infection, mice were sacrificed, and splenocytes were isolated to measure the antigen-specific CD8 T cell response by flow cytometry.

To analyze the percentages of antigen-specific CD8 T cell, splenocytes were stained with CD8α(53-6.7)-eFluor 450 (eBioscience, Cat# 48-0081-82), TCRβ(H57-597)-APC (BioLegend, Cat# 109212), CD44(IM7)-Percp-Cy5.5 (BioLegend, Cat# 103032), together with PE-conjugated H-2D^b^-gp^33–41^-tetramer (MBL, Cat# TB-5002-1), in the presence of Fc block in FACS buffer for 30 min at room temperature. Cells were washed twice with FACS buffer and analyzed by a LSR Fortessa cytometer (BD). Dead cells were excluded by DAPI staining.

To analyze the percentages of IFNγ^+^ CD8 T cells, splenocytes were stimulated with 0.2 μg/mL GP_33–41_ peptide (Chinapeptides) ex vivo for 5 h at 37 °C in the presence of GolgiSTOP (BD Pharmingen, Cat# 554724). Cell surface proteins were stained with CD8α(53-6.7)-PE (BioLegend, Cat# 100708) in the presence of Fc block and LIVE/DEAD Fixable Near-IR in FACS buffer at 4 °C for 15 min. Intracellular protein staining for IFNγ (BioLegend, Cat# 505806) was performed with the Cytofix/Cytoperm Plus Fixation/Permeabilization Kit according to the manufacturer’s instructions (BD Pharmingen, Cat# 554715). Cells were washed twice and analyzed by an LSR Fortessa cytometer (BD).

### *L. monocytogenes* infection

LM-OVA was generously provided by Ming O. Li’s lab at Memorial Sloan-Kettering Cancer Center. Mice were injected with 5000 colony-forming units of LM-OVA via tail vein. On day 7.5 post infection, mice were sacrificed, and splenocytes were isolated to measure antigen-specific CD8 T cell response by flow cytometry, following previously described methods. PE-conjugated H-2K^b^-OVA^257–264^-tetramer (MBL, Cat# TS-5001-1C) was used to measure antigen-specific CD8 T cells. Splenocytes were also stimulated with 10 ng/mL OVA^257–264^ peptide (SIINFEKL) (Chinapeptides, Cat# 138831-86-4) for 5 h ex vivo in the presence of GolgiSTOP to examine INFγ production by flow cytometry, following the methods described previously.

### Quantification and statistical analysis

The investigators were not blinded to the treatment groups. Data are presented as means ± SEM. The *P* values and number of replicates (*n*) are shown in figures or figure legends. For in vivo/ex vivo experiments, *n* represents the number of animals. For in vitro cell culture experiments, *n* indicates the number of independent experiments. GraphPad Prism 8.0 was used for statistical analysis. Paired or unpaired two-tailed Student’s *t*-test, one-way ANOVA or two-way ANOVA were used to evaluate the difference between groups. *P* < 0.05 was considered significant. All experiments were repeated independently at least twice with consistent results. Representative flow plots, immunoblots, and micrographs were selected from biological replicates.

## Supplementary information

Supplementary information

## Data Availability

The raw and processed data of scRNA-seq were deposited in the GEO database with the accession number GSE285373. This paper does not contain original code. Animal strains used in this study are available from The Jackson Laboratory and Cyagen Biosciences. Any additional information required to reanalyze the data reported in this paper is available from the authors upon request. All other data are available in the main text or Supplementary information.

## References

[1] Berridge, M. J., Lipp, P. & Bootman, M. D. The versatility and universality of calcium signalling. *Nat. Rev. Mol. Cell Biol.* **1**, 11–21 (2000). 11413485 10.1038/35036035

[2] Mekahli, D., Bultynck, G., Parys, J. B., De Smedt, H. & Missiaen, L. Endoplasmic-reticulum calcium depletion and disease. *Cold Spring Harb. Perspect. Biol.* **3**, a004317 (2011). 21441595 10.1101/cshperspect.a004317 PMC3098671

[3] Oakes, S. A. & Papa, F. R. The role of endoplasmic reticulum stress in human pathology. *Annu. Rev. Pathol.* **10**, 173–194 (2015). 25387057 10.1146/annurev-pathol-012513-104649 PMC5568783

[4] Schrank, S., Barrington, N. & Stutzmann, G. E. Calcium-handling defects and neurodegenerative disease. *Cold Spring Harb. Perspect. Biol.* **12**, a035212 (2020). 31427373 10.1101/cshperspect.a035212 PMC7328457

[5] Wang, W. A., Agellon, L. B. & Michalak, M. Organellar calcium handling in the cellular reticular network. *Cold Spring Harb. Perspect. Biol.* **11**, a038265 (2019). 31358518 10.1101/cshperspect.a038265 PMC6886452

[6] Wuytack, F., Raeymaekers, L. & Missiaen, L. Molecular physiology of the SERCA and SPCA pumps. *Cell Calcium* **32**, 279–305 (2002). 12543090 10.1016/s0143416002001847

[7] Hogan, P. G., Lewis, R. S. & Rao, A. Molecular basis of calcium signaling in lymphocytes: STIM and ORAI. *Annu. Rev. Immunol.* **28**, 491–533 (2010). 20307213 10.1146/annurev.immunol.021908.132550 PMC2861828

[8] Trebak, M. & Kinet, J. P. Calcium signalling in T cells. *Nat. Rev. Immunol.* **19**, 154–169 (2019). 30622345 10.1038/s41577-018-0110-7 PMC6788797

[9] Vaeth, M., Kahlfuss, S. & Feske, S. CRAC channels and calcium signaling in T cell-mediated immunity. *Trends Immunol* **41**, 878–901 (2020). 32711944 10.1016/j.it.2020.06.012 PMC7985820

[10] Martonosi, A. & Feretos, R. Sarcoplasmic reticulum. I. The uptake of Ca++ by sarcoplasmic reticulum fragments. *J. Biol. Chem* **239**, 648–658 (1964). 14169170

[11] Lewis, R. S. Store-operated calcium channels: new perspectives on mechanism and function. *Cold Spring Harb. Perspect. Biol.* **3**, a003970 (2011). 21791698 10.1101/cshperspect.a003970 PMC3225942

[12] Thastrup, O., Cullen, P. J., Drøbak, B. K., Hanley, M. R. & Dawson, A. P. Thapsigargin, a tumor promoter, discharges intracellular Ca2+ stores by specific inhibition of the endoplasmic reticulum Ca2(+)-ATPase. *Proc. Natl. Acad. Sci. USA* **87**, 2466–2470 (1990). 2138778 10.1073/pnas.87.7.2466 PMC53710

[13] Camello, C., Lomax, R., Petersen, O. H. & Tepikin, A. V. Calcium leak from intracellular stores-the enigma of calcium signalling. *Cell Calcium* **32**, 355–361 (2002). 12543095 10.1016/s0143416002001926

[14] Giunti, R., Gamberucci, A., Fulceri, R., Bánhegyi, G. & Benedetti, A. Both translocon and a cation channel are involved in the passive Ca2+ leak from the endoplasmic reticulum: a mechanistic study on rat liver microsomes. *Arch. Biochem. Biophys.* **462**, 115–121 (2007). 17481572 10.1016/j.abb.2007.03.039

[15] Sammels, E., Parys, J. B., Missiaen, L., De Smedt, H. & Bultynck, G. Intracellular Ca2+ storage in health and disease: a dynamic equilibrium. *Cell Calcium* **47**, 297–314 (2010). 20189643 10.1016/j.ceca.2010.02.001

[16] Hamilton, S. E. & Jameson, S. C. CD8 T cell quiescence revisited. *Trends Immunol* **33**, 224–230 (2012). 22361353 10.1016/j.it.2012.01.007 PMC3348359

[17] Bennett, T. J., Udupa, V. A. V. & Turner, S. J. Running to stand still: naive CD8(+) T cells actively maintain a program of quiescence. *Int. J. Mol. Sci.* **21**, 9773 (2020). 33371448 10.3390/ijms21249773 PMC7767439

[18] Surh, C. D. & Sprent, J. Homeostasis of naive and memory T cells. *Immunity* **29**, 848–862 (2008). 19100699 10.1016/j.immuni.2008.11.002

[19] Josefowicz, S. Z., Lu, L. F. & Rudensky, A. Y. Regulatory T cells: mechanisms of differentiation and function. *Annu. Rev. Immunol.* **30**, 531–564 (2012). 22224781 10.1146/annurev.immunol.25.022106.141623 PMC6066374

[20] Li, M. O. & Flavell, R. A. TGF-β: A master of all T cell trades. *Cell* **134**, 392–404 (2008). 18692464 10.1016/j.cell.2008.07.025 PMC3677783

[21] ElTanbouly, M. A. & Noelle, R. J. Rethinking peripheral T cell tolerance: checkpoints across a T cell’s journey. *Nat. Rev. Immunol.* **21**, 257–267 (2021). 33077935 10.1038/s41577-020-00454-2 PMC12536352

[22] Chapman, N. M., Boothby, M. R. & Chi, H. Metabolic coordination of T cell quiescence and activation. *Nat. Rev. Immunol.* **20**, 55–70 (2020). 31406325 10.1038/s41577-019-0203-y

[23] Buck, M. D., Sowell, R. T., Kaech, S. M. & Pearce, E. L. Metabolic instruction of immunity. *Cell* **169**, 570–586 (2017). 28475890 10.1016/j.cell.2017.04.004 PMC5648021

[24] MacIver, N. J., Michalek, R. D. & Rathmell, J. C. Metabolic regulation of T lymphocytes. *Annu. Rev. Immunol.* **31**, 259–283 (2013). 23298210 10.1146/annurev-immunol-032712-095956 PMC3606674

[25] Blagih, J., Krawczyk, C. M. & Jones, R. G. LKB1 and AMPK: central regulators of lymphocyte metabolism and function. *Immunol. Rev.* **249**, 59–71 (2012). 22889215 10.1111/j.1600-065X.2012.01157.x

[26] Peng, M. & Li, M. O. Metabolism along the life journey of T cells. *Life Metab.* **2**, load002 (2023). 37305096 10.1093/lifemeta/load002 PMC10256237

[27] Morita, K. et al. Genome-wide CRISPR screen identifies TMEM41B as a gene required for autophagosome formation. *J. Cell Biol.* **217**, 3817–3828 (2018). 30093494 10.1083/jcb.201804132 PMC6219718

[28] Shoemaker, C. J. et al. CRISPR screening using an expanded toolkit of autophagy reporters identifies TMEM41B as a novel autophagy factor. *PLoS Biol* **17**, e2007044 (2019). 30933966 10.1371/journal.pbio.2007044 PMC6459555

[29] Hoffmann, H. H. et al. TMEM41B is a pan-flavivirus host factor. *Cell* **184**, 133–148.e20 (2021). 33338421 10.1016/j.cell.2020.12.005 PMC7954666

[30] Sun, L. et al. Genome-scale CRISPR screen identifies TMEM41B as a multi-function host factor required for coronavirus replication. *PLoS Pathog.* **17**, e1010113 (2021). 34871328 10.1371/journal.ppat.1010113 PMC8675922

[31] Trimarco, J. D. et al. TMEM41B is a host factor required for the replication of diverse coronaviruses including SARS-CoV-2. *PLoS Pathog* **17**, e1009599 (2021). 34043740 10.1371/journal.ppat.1009599 PMC8189496

[32] Huang, D. et al. TMEM41B acts as an ER scramblase required for lipoprotein biogenesis and lipid homeostasis. *Cell Metab.* **33**, 1655–1670.e8 (2021). 34015269 10.1016/j.cmet.2021.05.006

[33] Li, Y. E. et al. TMEM41B and VMP1 are scramblases and regulate the distribution of cholesterol and phosphatidylserine. *J. Cell Biol.* **220**, e202103105 (2021). 33929485 10.1083/jcb.202103105 PMC8077175

[34] Ji, M. et al. VMP1 and TMEM41B are essential for DMV formation during beta-coronavirus infection. *J. Cell Biol.* **221**, e202112081 (2022). 35536318 10.1083/jcb.202112081 PMC9097365

[35] Yousefi, M. et al. TMEM41B and VMP1 modulate cellular lipid and energy metabolism for facilitating dengue virus infection. *PLoS Pathog.* **18**, e1010763 (2022). 35939522 10.1371/journal.ppat.1010763 PMC9387935

[36] Ji, M., Li, M., Sun, L., Deng, H. & Zhao, Y. G. DMV biogenesis during beta-coronavirus infection requires autophagy proteins VMP1 and TMEM41B. *Autophagy* **19**, 737–738 (2023). 35900889 10.1080/15548627.2022.2103783 PMC9851257

[37] Liu, Y. et al. VMP1 prevents Ca2+ overload in endoplasmic reticulum and maintains naive T cell survival. *J. Exp. Med.* **220**, e20221068 (2023). 36971758 10.1084/jem.20221068 PMC10060355

[38] Suzuki, J. et al. Imaging intraorganellar Ca2+ at subcellular resolution using CEPIA. *Nat. Commun.* **5**, 4153 (2014). 24923787 10.1038/ncomms5153 PMC4082642

[39] Arakel, E. C. & Schwappach, B. Formation of COPI-coated vesicles at a glance. *J. Cell Sci.* **131**, jcs209890 (2018). 29535154 10.1242/jcs.209890

[40] Owsianik, G., Talavera, K., Voets, T. & Nilius, B. Permeation and selectivity of TRP channels. *Annu. Rev. Physiol.* **68**, 685–717 (2006). 16460288 10.1146/annurev.physiol.68.040204.101406

[41] Kuo, C. T., Veselits, M. L. & Leiden, J. M. LKLF: A transcriptional regulator of single-positive T cell quiescence and survival. *Science* **277**, 1986–1990 (1997). 9302292 10.1126/science.277.5334.1986

[42] Chi, H. Regulation and function of mTOR signalling in T cell fate decisions. *Nat. Rev. Immunol.* **12**, 325–338 (2012). 22517423 10.1038/nri3198 PMC3417069

[43] Kim, D. H. et al. MTOR interacts with Raptor to form a nutrient-sensitive complex that signals to the cell growth machinery. *Cell* **110**, 163–175 (2002). 12150925 10.1016/s0092-8674(02)00808-5

[44] Voisinne, G., de Peredo, A. G. & Roncagalli, R. CD5, an undercover regulator of TCR signaling. *Front. Immunol.* **9**, 2900 (2018). 30581443 10.3389/fimmu.2018.02900 PMC6292949

[45] Yusuf, I. & Fruman, D. A. Regulation of quiescence in lymphocytes. *Trends Immunol.* **24**, 380–386 (2003). 12860529 10.1016/s1471-4906(03)00141-8

[46] ElTanbouly, M. A. et al. VISTA is a checkpoint regulator for naive T cell quiescence and peripheral tolerance. *Science* **367**, eaay0524 (2020). 31949051 10.1126/science.aay0524 PMC7391053

[47] Whitlock, J. M. & Hartzell, H. C. Anoctamins/TMEM16 proteins: chloride channels flirting with lipids and extracellular vesicles. *Annu. Rev. Physiol.* **79**, 119–143 (2017). 27860832 10.1146/annurev-physiol-022516-034031 PMC5556385

[48] Okawa, F. et al. Evolution and insights into the structure and function of the DedA superfamily containing TMEM41B and VMP1. *J. Cell Sci.* **134**, jcs255877 (2021). 33771928 10.1242/jcs.255877

[49] Braga, L. et al. Drugs that inhibit TMEM16 proteins block SARS-CoV-2 spike-induced syncytia. *Nature* **594**, 88–93 (2021). 33827113 10.1038/s41586-021-03491-6 PMC7611055

[50] Zheng, Q. X. et al. Calcium transients on the ER surface trigger liquid-liquid phase separation of FIP200 to specify autophagosome initiation sites. *Cell* **185**, 4082–4098.e22 (2022). 36198318 10.1016/j.cell.2022.09.001

[51] Yang, K., Neale, G., Green, D. R., He, W. F. & Chi, H. B. The tumor suppressor Tsc1 enforces quiescence of naive T cells to promote immune homeostasis and function. *Nat. Immunol.* **12**, 888–897 (2011). 21765414 10.1038/ni.2068 PMC3158818

[52] Blanco, D. B. et al. PTEN directs developmental and metabolic signaling for innate-like T cell fate and tissue homeostasis. *Nat. Cell Biol.* **24**, 1642–1654 (2022). 36302969 10.1038/s41556-022-01011-w PMC10080469

[53] Ouyang, W. M., Beckett, O., Flavell, R. A. & Li, M. O. An essential role of the forkhead-box transcription factor Foxo1 in control of T cell homeostasis and tolerance. *Immunity* **30**, 358–371 (2009). 19285438 10.1016/j.immuni.2009.02.003 PMC2692529

[54] Da Silva, J. D. et al. Loss of the orthologue of a downstream target of SMN, leads to abnormalities in sensorimotor integration. *Mol. Neurobiol.* **57**, 1553–1569 (2020). 31797327 10.1007/s12035-019-01833-0

[55] Simon, C. M. et al. Stasimon contributes to the loss of sensory synapses and motor neuron death in a mouse model of spinal muscular atrophy. *Cell Rep.* **29**, 3885–3901.e5 (2019). 31851921 10.1016/j.celrep.2019.11.058 PMC6956708

