# Supplementary material for: TMEM41B is an endoplasmic reticulum Ca2+ release channel maintaining naive T cell quiescence and responsiveness
Source: Cell Discov. 2025 Mar 4;11:18. doi: 10.1038/s41421-024-00766-w (PMC11880246; doi:10.1038/s41421-024-00766-w)
Supplement: Supplementary file 1 — Supplementary information [file 41421_2024_766_MOESM1_ESM.pdf]

Supplementary information

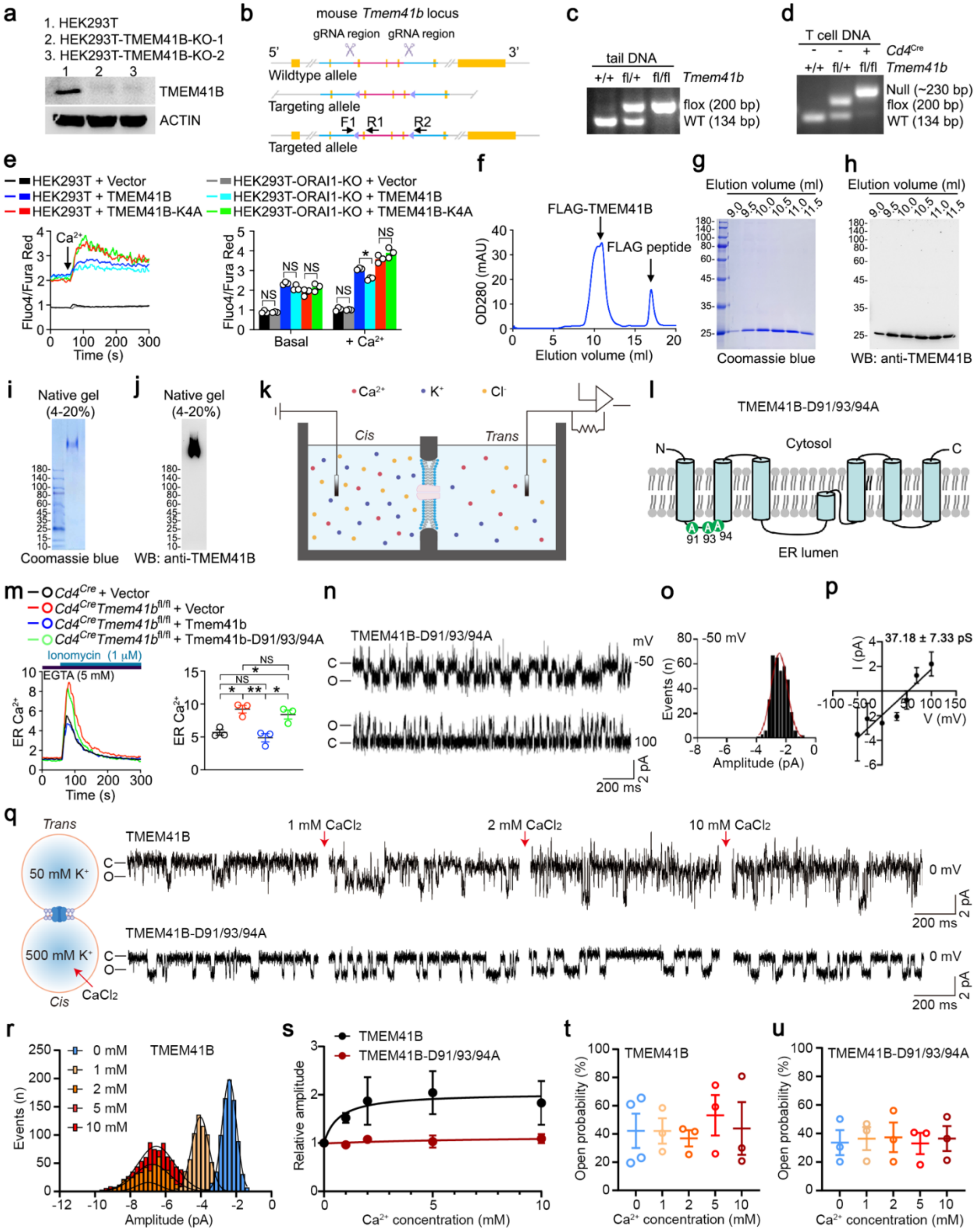

Supplementary Fig. S1 Additional supporting data related to Figs. 1-3

a Immunoblotting was performed to detect TMEM41B expression in wild-type and TMEM41B monoclonal-knockout (KO) HEK293T cells. ACTIN was utilized as a loading control. Representative blots from 2 independent experiments are presented.

b Schematic representation illustrating the gene targeting strategy employed for the generation of a *Tmem41b* flox allele.

c Genotyping analysis of DNA extracted from mouse tail samples with indicated genotypes.

d Genotyping analysis of DNA extracted from T cells of mice with indicated genotypes.

e Flow cytometry analysis of Ca^{2+} influx in ORAI1-KO HEK293T cells transfected with indicated plasmids. Representative plots and statistical analysis from one of three independent experiments are shown (n = 3 samples).

f A representative plot of size exclusion chromatography (SEC) of purified TMEM41B.

g Coomassie blue staining of TMEM41B from indicated elution volume of SEC by SDS-PAGE.

h Immunoblotting of TMEM41B from indicated elution volume of SEC by SDS-PAGE.

i Coomassie blue staining showing TMEM41B separation by native gel electrophoresis.

j Immunoblotting showing TMEM41B separation by native gel electrophoresis.

k A carton of planar lipid bilayer work station (BLM Workstation).

l Putative topology model of TMEM41B protein, with D91/93/94 mutations indicated. The mutated protein was named as TMEM41B-D91/93/94A.

m Flow cytometry analysis of ER Ca^{2+} store in TMEM41B-deficient CD4 T cells retrovirally expressing indicated proteins, 40 days post-resting in recipient mice. Representative plots and statistical analysis are shown (n = 3 mice).

n Representative single channel currents of TMEM41B-D91/93/94A in 500: 50 KCl solution at indicated voltage.

o All-point current histograms corresponding to the trace in (n).

p I-V curve of TMEM41B in solution of (n).

q TMEM41B and TMEM41B-D91/93/94A channels were recorded at 0 mV with indicated Ca^{2+} concentrations added to *Cis* side in 50:500 mM KCl solutions (*Trans: Cis*). Red arrow indicates when $CaCl_2$ was added.

r All-points amplitude histograms of TMEM41B were constructed from panel (q). The data shown are from a single experiment. The experiment was repeated three more times with similar results.

s Normalized to the value without Ca^{2+}. Relative amplitude of TMEM41B and TMEM41B-D91/93/94A in (q). The values are normalized to the value without Ca^{2+} (n $\geqslant$ 3 independent experiments).

t Relative open probability of TMEM41B in (q) (n $\geqslant$ 3 independent experiments).

u Relative open probability of TMEM41B-D91/93/94A in (q) (n $\geqslant$ 3 independent experiments).

Data represent mean ± SEM, two-tailed unpaired t-test in e, one-way ANOVA in m, * $p< 0.05$, ** $p< 0.01$, NS, not significant.

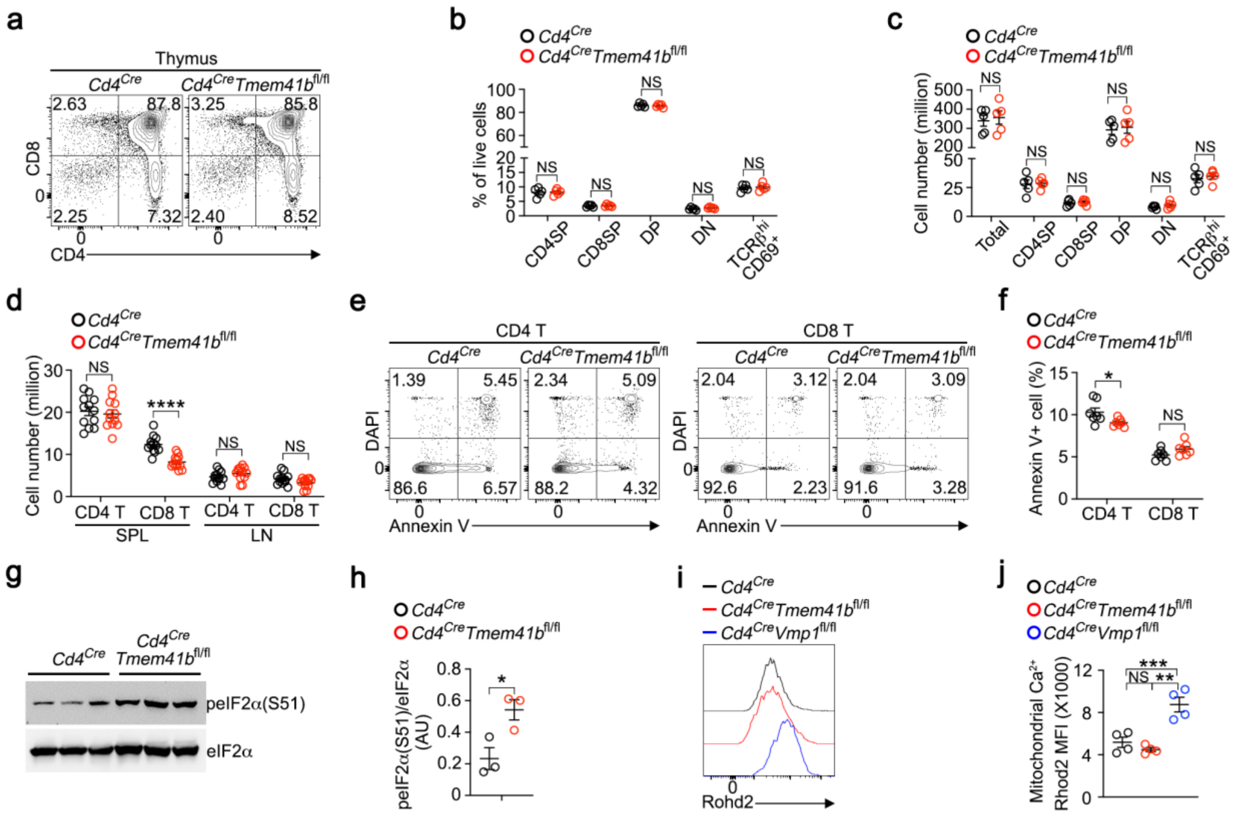

Supplementary Fig. S2 Additional data related to Fig. 4

a-c Flow cytometry analysis of thymocytes from control (*Cd4*^{Cre}) and TMEM41B-deficient (*Cd4*^{Cre}*Tmem41b*$^{fl/fl}$) mice. Representative plots (a) and statistical analysis are shown, indicating the percentages (b) and cell numbers (c) of CD4^{+}CD8^{-} (CD4SP), CD4^{-}CD8^{+} (CD8SP), CD4^{+}CD8^{+} (DP), CD4^{-}CD8^{-} (DN) cells (n = 5 mice).

d T cell number in spleen (SPL) and peripheral lymph nodes (pLN) of control and TMEM41B-deficient mice (n = 12 mice).

e and f Flow cytometry analysis of T cell apoptosis. Representative plots (e) and statistical analysis (f) are shown (n = 7 mice).

g and h Immunoblotting of eIF2α and its phosphorylation form (peIF2α-S51) in freshly isolated T cells from control and TMEM41B-deficient mice. Representative plots (g) and statistical analysis (h) are shown. Each lane represents an individual mouse (n = 3 mice).

i and j Flow cytometry analysis of mitochondrial Ca^{2+} levels in control, TMEM41B-deficient, and VMP1-deficient CD4 T cells. Representative plots (i) and statistical analysis (j) are shown (n = 4 mice).

Data represent mean ± SEM, two-tailed unpaired t-test in b, c, d, f and h, one-way ANOVA in j, * $p< 0.05$, ** $p< 0.01$, *** $p< 0.001$, **** $p< 0.0001$, NS, not significant.

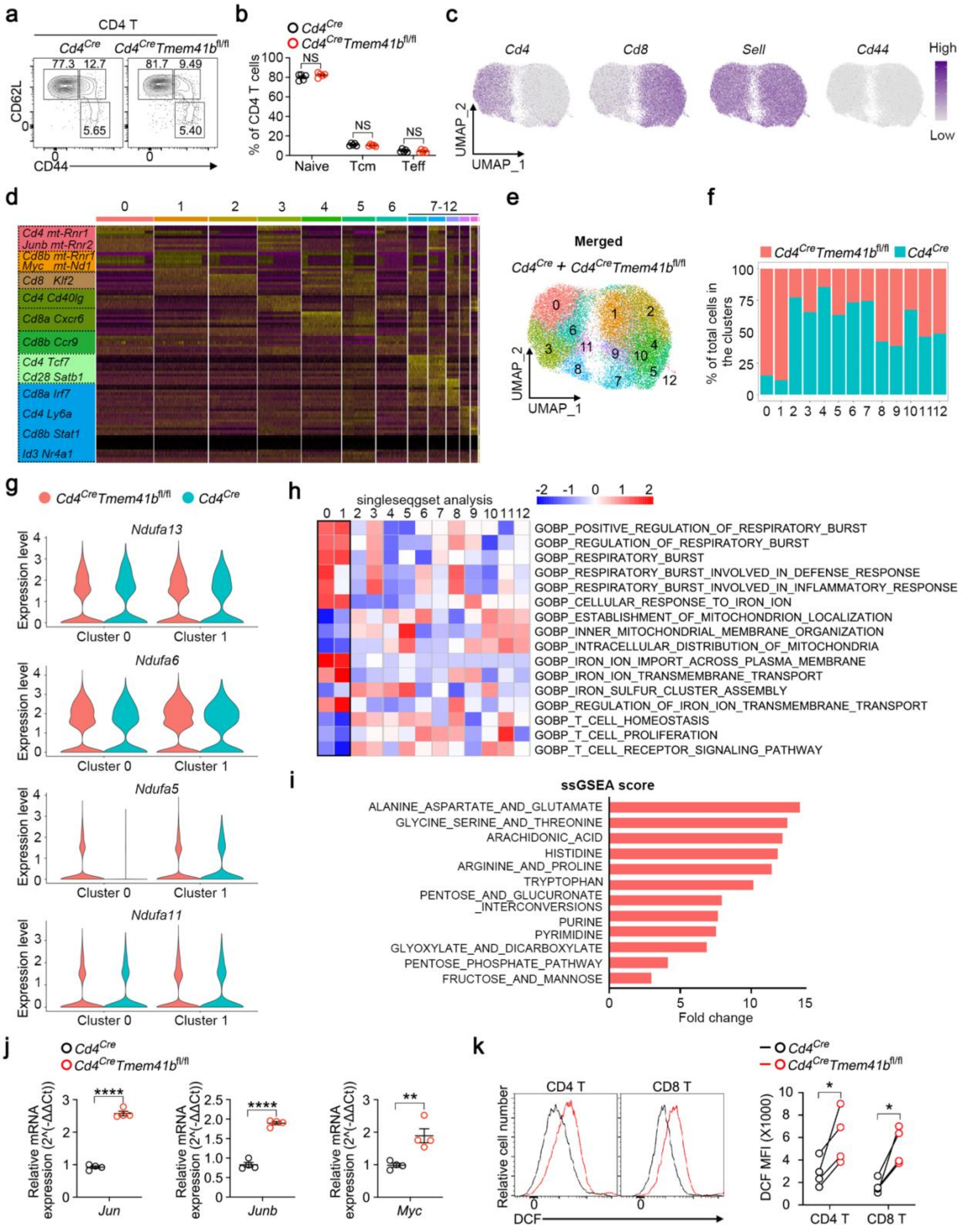

Supplementary Fig. S3 Additional data related to Fig. 4

a and b Flow cytometry analysis was conducted to assess the activation status (CD44 vs CD62L) of CD4 T cells from peripheral lymph nodes (pLN) of control (*Cd4*^{Cre}) and TMEM41B-deficient (*Cd4*^{Cre}*Tmem41b*$^{\mathrm{fl/fl}}$) mice (6 ~ 8-week-old). Representative plots (a) and statistical analysis (b) are shown (n = 5 mice).

c Uniform manifold approximation and projection (UMAP) plots illustrating the distribution of pooled naive T cells from both control and TMEM41B-deficient mice. Each dot represents an individual cell. The depth of color corresponds to the level of gene expression.

d Heatmap visualization depicting 13 clusters, with defining genes for each cluster displayed.

e Unsupervised clustering of pooled naive T cells, including both control and TMEM41B-deficient cells, categorized by cluster classification. Each cluster is represented by a distinct color.

f The percentages of control and TMEM41B-deficeint naive T cells within each cluster shown in panel (e).

g Violin plots showing gene expression in Cluster 0 and Cluster 1 for control and TMEM41B-deficient naive T cells.

h Gene-set enrichment analysis (GSEA) of all clusters in pooled naive T cells from both control and TMEM41B-deficient mice by SingleSeqGset analysis method.

i Gene-set enrichment analysis (GSEA) of upregulated gene sets associated with amino acid metabolism in TMEM41B-deficient naive T cells compared to control naive T cells, conducted using the single-sample GSEA (ssGSEA) method.

j Quantitative PCR (qPCR) analysis was performed to measure the mRNA expression levels of *Jun*, *Junb*, and *Myc* in naive T cells from control and TMEM41B-deficient mice, with *Hprt* used for normalization. Representative data from one of three independent experiments are shown (n = 4 replicates).

k Flow cytometry analysis of reactive oxygen species (ROS) in control and TMEM41B-deficient naive T cells. Representative plots and statistical analysis are shown. (n = 4 mice)

Data represent mean ± SEM, two-tailed unpaired t-test in b and j, paired t-test in k, * p< 0.05, ** p< 0.01, **** p< 0.0001, NS, not significant.

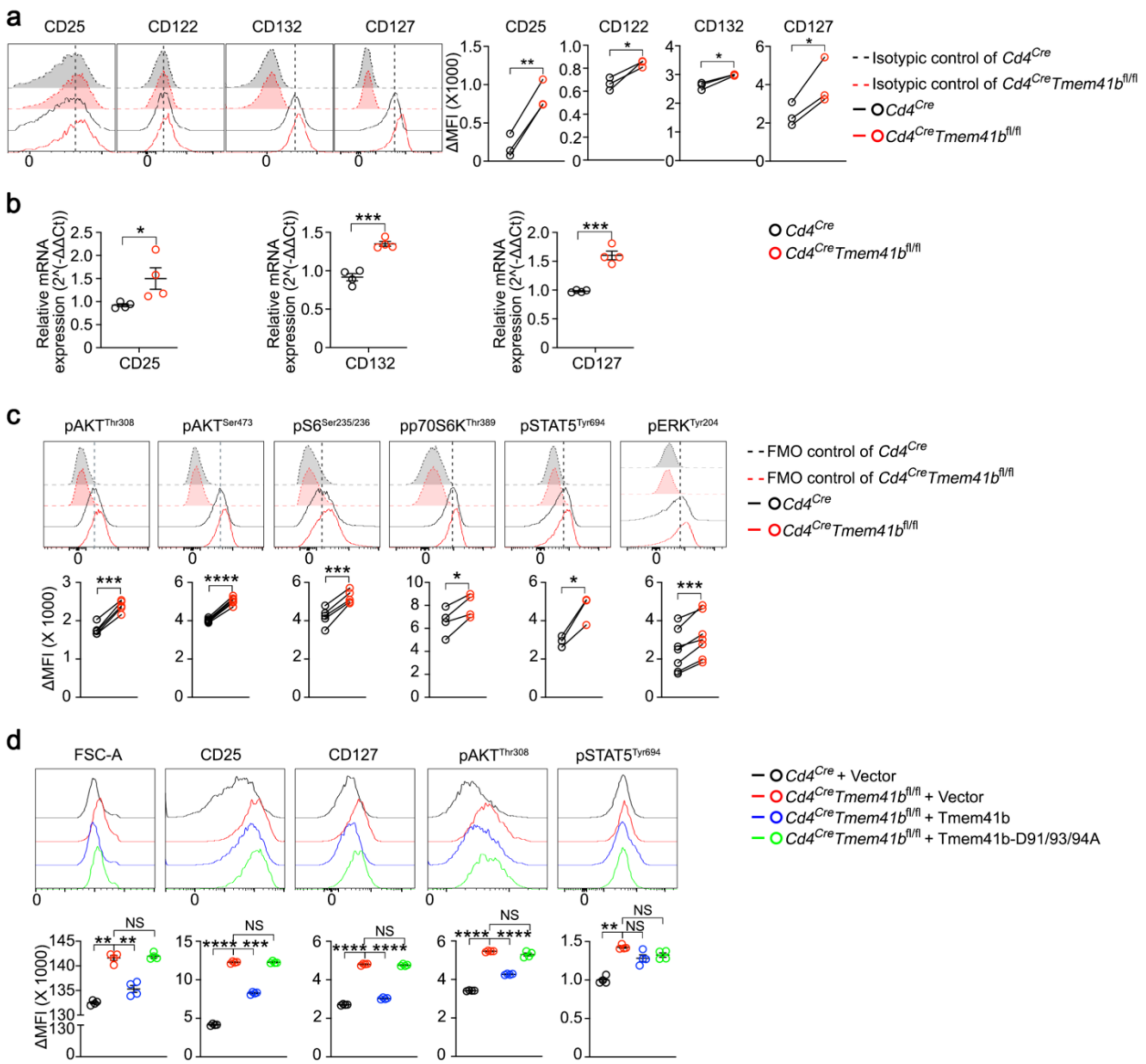

Supplementary Fig. S4 Additional data related to Fig. 5

a Flow cytometry analysis was conducted to assess the expression levels of indicated proteins in naive CD4 T cells isolated from peripheral lymph nodes (pLN) of control (*Cd4*^{Cre}) and TMEM41B-deficient (*Cd4*^{Cre}*Tmem41b*$^{\text{fl/fl}}$) mice. ΔMFI represents the MFI of the target marker after subtracting the MFI of the isotype control. Representative plots and statistical analysis are shown (n = 3 mice).

b Quantitative PCR (qPCR) was used to measure the mRNA expression levels of CD25 (IL-2Rα), CD132 (IL-2Rγ), and CD127 (IL-7Rα) in naive T cells, with *Hprt* serving as the reference gene for normalization. Representative statistical analysis from one of three independent experiments is shown (n = 4 replicates).

c Flow cytometry analysis was conducted to assess the phosphorylation levels of indicated proteins in naive CD4 T cells isolated from pLN of control and TMEM41B-deficient mice. Fluorescence minus one (FMO) control was used to ensure accurate measurement. ΔMFI represents the MFI of the target marker after subtracting the MFI of the FMO control. Representative plots and statistical analysis from one of three independent experiments are shown (n = 3 ~ 8 mice).

d Activated CD8 T cells were transduced with retroviral constructs as indicated. Flow cytometry analysis was performed 72 hours post-transduction to measure cell size, CD25/CD127 expression, and phosphorylation levels of AKT and STAT5. ΔMFI represents the MFI of the target marker after subtracting the MFI of the FMO control. Representative plots and statistical analysis from one of three independent experiments are shown (n = 4 samples).

Data represent mean ± SEM, two-tailed paired t-test in a and c, unpaired t-test in b, one-way ANOVA in d, * $p < 0.05$, ** $p < 0.01$, *** $p< 0.001$, **** $p < 0.0001$, NS, not significant.

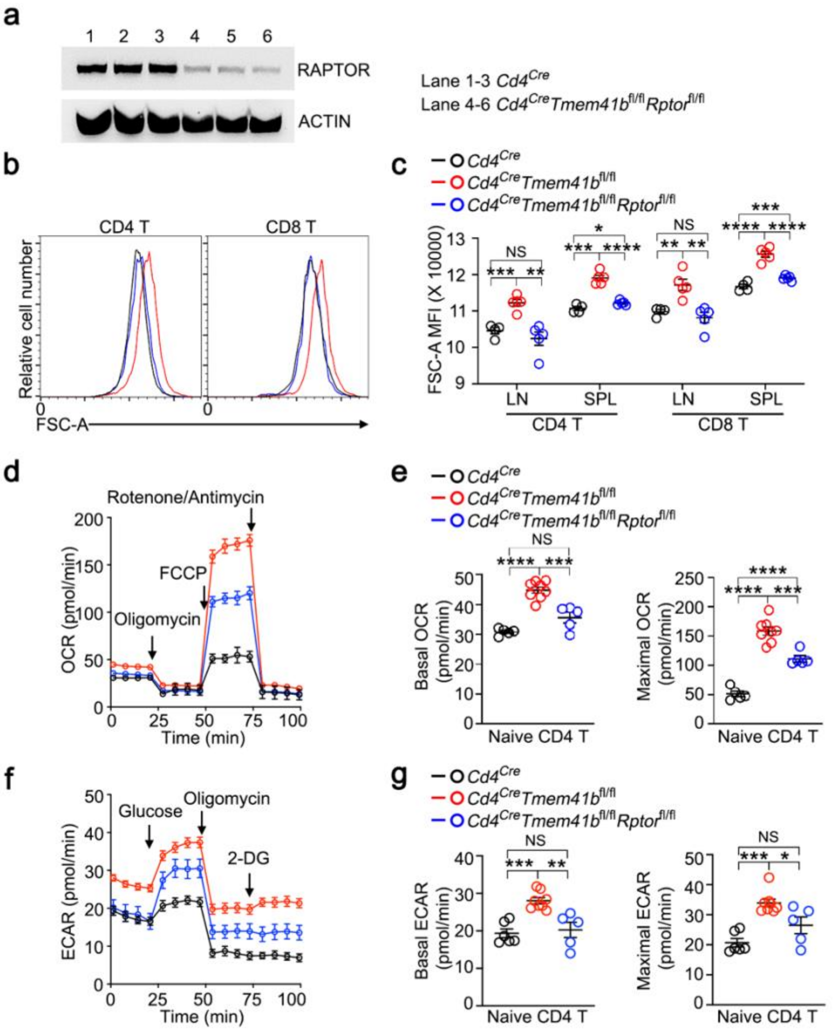

Supplementary Fig. S5 Additional data related to Fig. 5

a Immunoblot analysis of RAPTOR expression in total T cells isolated from control (*Cd4*^{Cre}) and TMEM41B-RAPTOR double knockout (DKO) (*Cd4*^{Cre}*Tmem41b*$^{\text{fl/fl}}$*Rptor*$^{\text{fl/fl}}$) mice. Each lane represents an individual mouse.

b and c Flow cytometry analysis of cell size in naive T cells isolated from control, and DKO mice. Representative plots (b) and statistical analysis (c) are shown (n = 4 or 5 mice).

d and e Oxygen consumption rate (OCR) of naive CD4 T cells from indicated genotypes were measured with a Mito stress test kit. Representative plots (d) and calculated basal

and maximal OCR (e) from one of three independent experiments are shown (n = 5 or 8 replicates for each group).

f and g Extracellular acidification rate (ECAR) of naive CD4 T cells with indicated genotypes were measured with a Glycolysis stress test kit. Representative plots (f) and calculated basal and maximal ECAR (g) from one of three independent experiments are shown (n = 5, 6 or 8 replicates for each group).

Data represent mean ± SEM, one-way ANOVA in c, e and g, * $p < 0.05$, ** $p < 0.01$, *** $p < 0.001$, **** $p < 0.0001$, NS, not significant.

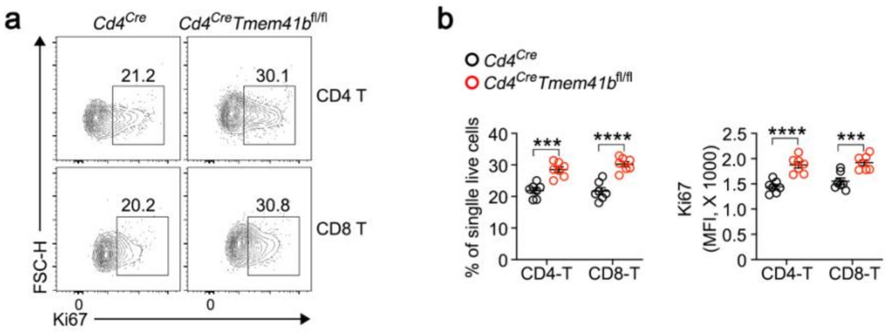

Supplementary Fig. S6 Additional data related to Fig. 7

a and b Freshly isolated T cells from control (*Cd4*^{Cre}) and TMEM41B-deficient (*Cd4*^{Cre}*Tmem41b*$^{\mathrm{fl/fl}}$) mice were stained for the proliferation marker Ki67 and analyzed via flow cytometry. Representative plots are shown in panel (a). Statistical analysis of the percentage of Ki67-positive T cells and the mean fluorescence intensity (MFI) of Ki67 is presented in panel (b) (n = 7 mice).

Data are presented as mean ± SEM. Statistical significance was determined using a two-tailed unpaired t-test, *** p< 0.001, **** p< 0.0001.
